# A multifunctional platform with single-NIR-laser-triggered photothermal and NO release for synergistic therapy against multidrug-resistant Gram-negative bacteria and their biofilms

**DOI:** 10.1186/s12951-020-00614-5

**Published:** 2020-04-15

**Authors:** Baohua Zhao, He Wang, Wenjing Dong, Shaowen Cheng, Haisheng Li, Jianglin Tan, Junyi Zhou, Weifeng He, Lanlan Li, Jianxiang Zhang, Gaoxing Luo, Wei Qian

**Affiliations:** 1 Institute of Burn Research, State Key Laboratory of Trauma, Burn and Combined Injury, Key Laboratory of Disease Proteomics of Chongqing, Southwest Hospital, Third Military Medical University (Army Medical University), Chongqing, 400038 China; 2 Department of Trauma Centre, The First Affiliated Hospital, Hainan Medical University, Haikou, 570102 Hainan China; 3 Department of Pharmaceutics, College of Pharmacy, Third Military Medical University (Army Medical University), Chongqing, 400038 China

**Keywords:** Graphene, Single-NIR-laser-triggered, Photothermal, NO release, Synergistic, Multidrug-resistant Gram-negative bacteria, Biofilms

## Abstract

**Background:**

Infectious diseases caused by multidrug-resistant (MDR) bacteria, especially MDR Gram-negative strains, have become a global public health challenge. Multifunctional nanomaterials for controlling MDR bacterial infections via eradication of planktonic bacteria and their biofilms are of great interest.

**Results:**

In this study, we developed a multifunctional platform (TG-NO-B) with single NIR laser-triggered PTT and NO release for synergistic therapy against MDR Gram-negative bacteria and their biofilms. When located at the infected sites, TG-NO-B was able to selectively bind to the surfaces of Gram-negative bacterial cells and their biofilm matrix through covalent coupling between the BA groups of TG-NO-B and the bacterial LPS units, which could greatly improve the antibacterial efficiency, and reduce side damages to ambient normal tissues. Upon single NIR laser irradiation, TG-NO-B could generate hyperthermia and simultaneously release NO, which would synergistically disrupt bacterial cell membrane, further cause leakage and damage of intracellular components, and finally induce bacteria death. On one hand, the combination of NO and PTT could largely improve the antibacterial efficiency. On the other hand, the bacterial cell membrane damage could improve the permeability and sensitivity to heat, decrease the photothermal temperature and avoid damages caused by high temperature. Moreover, TG-NO-B could be effectively utilized for synergistic therapy against the in vivo infections of MDR Gram-negative bacteria and their biofilms and accelerate wound healing as well as exhibit excellent biocompatibility both in vitro and in vivo.

**Conclusions:**

Our study demonstrates that TG-NO-B can be considered as a promising alternative for treating infections caused by MDR Gram-negative bacteria and their biofilms.

## Introduction

Bacterial infection associated diseases have become one of the major public health issues and attracted worldwide concerns [1]. Infections caused by multidrug-resistant (MDR) strains, particularly MDR Gram-negative bacteria, e.g., *Pseudomonas aeruginosa (Pa)*, *Klebsiella pneumonia (Kp)*, *Acinetobacter baumannii* (*Ab*), etc., have increased steadily over the last decade and are associated with high rates of morbidity and mortality [2]. The conventional treatment for bacterial infections is the use of antibiotics, but the efficiency of antibiotics is threatened by the increased antibiotic resistance of bacteria [3]. It is worthy of note that antibiotic resistance not only originates from the transformation of the structure and gene mutations of planktonic bacteria but also results from the formation of bacterial biofilms [4]. Almost 90% microorganisms can form biofilms, in which bacterial cells are frequently embedded in a self-produced matrix of extracellular polymeric substances (EPS) which basically consist of polysaccharides, proteins, lipids and extracellular DNA [5, 6]. The EPS matrix can act as a protective barrier that confers resistance to nonspecific and specific host defenses during infection and prevents the penetration of antibacterial agents [7, 8]. The high resistance towards host immune response and traditional antibacterial therapy makes associated infections more intractable and challenging. In response to this urgent challenge, it is imperative to develop novel strategies to fight against MDR bacteria and biofilm infections.

Recently, photothermal therapy (PTT) using a NIR laser-absorbing nanomaterial is recognized as one of the most promising strategies for combating bacterial infections [9, 10]. Under NIR laser irradiation, the photothermal nanomaterial converts light energy into heat and elevates the local temperature, which can induce the bacterial cell membrane destruction and protein denaturation and the dispersion of biofilms [9, 11–13]. PTT offers a lot of advantages, e.g. deep tissue penetration, little light absorption and spatiotemporal controllability [14]. Moreover, localized NIR irradiation can promote blood circulation and relieve inflammation of tissues [15], which is beneficial for wound healing. Up to now, various types of photothermal nanomaterials have been explored for applications in the antibacterial therapy, including carbon-based nanoparticles [9, 16], metal nanoparticles [12, 17] and polymeric nanoparticles [10, 18]. Among of them, graphene, as a monolayer of carbon atoms packed into a dense honeycomb crystal structure, has drawn much attention owing to its special two-dimensional structure, strong mechanical property, high photothermal conversion efficiency and excellent biocompatibility [9, 19]. In 2013, Wu et al. [16] fabricated a magnetic reduced graphene oxide/glutaraldehyde nanocomposite, which could effectively kill Gram-positive *Staphylococcus aureus* (*S. aureus*) and Gram-negative *Escherichia coli* (*E. coli*) when exposed to NIR laser irradiation. In 2018, our group [9] also prepared a surface-adaptive nanomaterial based on chitosan and carboxyl graphene, which could induce thermal ablation of *S. aureus*, *E. coli* and Methicillin-resistant *S. aureus* (*MRSA*). Nevertheless, previous PTTs based on nanomaterials mainly focused on the eradication of nondrug-resistant strains and *MRSA* bacteria. Thus, an ideal and special antibacterial system capable of eliminating MDR Gram-negative bacteria and biofilms is indeed needed to be explored. Furthermore, there are still some drawbacks that hinder the practical application of PTT. For example, photothermal nanoagents themselves cannot selectively bind to pathogenic bacteria or biofilms, which may inevitably cause undesirable damage the ambient healthy tissues [9, 19]. Besides, current working temperatures of PTT are too high (55–60 °C), which can potentially burn the normal skin and other tissues [20]. Moreover, monotherapy is not as effective as expected in treating bacterial infections [21]. Therefore, these issues highlight the demand for alternative system with effective targeting or combinational therapy and low-temperature PTT.

Nitric oxide (NO), as an endogenously produced molecule which plays an important role in physiological and pathophysiological processes such as wound healing and immune responses, has attracted much attention [22]. Recently, NO has been recognized as a broad-spectrum bactericidal agent which is capable of killing bacteria, especially for drug-resistant bacteria [8, 15]. NO and its byproducts like nitrogen dioxide, dinitrogen trioxide and peroxynitrite can cause nitrosative and oxidative stresses on bacteria, resulting in lipid peroxidation, rupture of bacterial cell membranes, DNA cleavage and protein dysfunction [15, 22]. In particular, unlike conventional antibacterial agents, NO also promotes wound healing via enhancing epidermal stem cell de-adhesion and proliferation, and increasing myofibroblasts and collagen production during skin reconstruction [23, 24]. Therefore, we have reasons to believe that the combination of NO and PTT will improve the antibacterial activity and bring more benefits. However, the short half-life of NO and the lack of suitable vehicles for NO storage and delivery greatly imped the development of NO-based therapeutic platforms for antibacterial applications [15, 25]. Furthermore, the realization of precisely controlled NO delivery and release also needs to be solved urgently. Nowadays, substantial efforts have been made to explore various types of NO donors and their innovative delivery nanovehicles as therapeutic platforms by modulating exogenous stimuli like light, pH, heat and enzymes [26, 27]. Among these, photo-triggered NO releasing platforms have become the most striking due to the permission of precise control of location, timing and dosage [28]. Nevertheless, most of the previously reported platforms are based on UV or visible light, and their therapeutic effects are strongly influenced by the shallow tissue penetration and toxic side effects resulting from short-wavelength light [29]. In contrast, near-infrared (NIR) in the range of 780–1100 nm may be a suitable wavelength region due to its deeper tissue penetration, less absorption by tissues and less damage to ambient tissues [9]. Some researchers have tried to use two-photon excitation with NIR light to realize NO release, but relatively high-power lasers are required, thereby causing damages to skin or other tissues [30–32]. To date, constructing novel NIR-light-triggered NO-releasing platforms are still in its infancy. Consequently, based on the antibacterial potential of NO, the design requirement of NO controlled-releasing platforms, and the large surface area and the high NIR photothermal effect of graphene as a nanocarrier and photothermal agent, we conceive a multifunctional platform which integrate graphene-based PTT and a photothermally sensitive NO donor. We anticipate that the multifunctional platform will overcome the drawbacks of PPT or NO alone. If the nanoplatform is capable of binding specifically to bacteria or biofilms, it will further improve the accuracy of PTT and NO releasing. Upon NIR laser irradiation, the NIR-triggered hyperthermia and NO releasing can disrupt the bacterial cell membrane, further cause leakage of intracellular components, and finally induce bacteria death. At the same time, the bacterial cell membrane damage can enhance the permeability and sensitivity to heat and lower the hyperthermia temperature [20]. Furthermore, the hyperthermia can also promote NO release, which may further damage the bacterial cells.

Boronic acid (BA), a boron center bearing three hydroxyl groups via carbon-boron bonds, can covalently bind to diol-containing saccharides and form boronic esters [33, 34]. Since a high level of lipopolysaccharides (LPS) with cis-diol groups are on Gram-negative bacterial cell surfaces [33], BA and its derivatives have been successfully used as a recognition molecule alternative for bacteria detection. Recently, Zheng et al. [33] fabricated a simple, rapid and cost-effective colorimetric assay for Gram-negative bacteria monitoring based on the 4-mercaptophenylboronic acid functionalized silver nanoparticles, which could specially absorb onto the surfaces of Gram-negative bacteria cells rather than Gram-positive ones. Galstyan et al. [35] developed an innovative strategy for targeting polysaccharides found on the Gram-negative bacterial cell envelop and the biofilm matrix using the boronic acid functionalized and highly effective photosensitizer silicon (IV) phthalocyanine, which was successful in treating planktonic cultures and biofilms of Gram-negative *E. coli*. Moreover, thus far, lack of obvious toxicity, in vivo stability and ease of handling have made boronic acid functionalized compounds or materials increasingly attractive for medical applications [36].

**Fig. 1:**
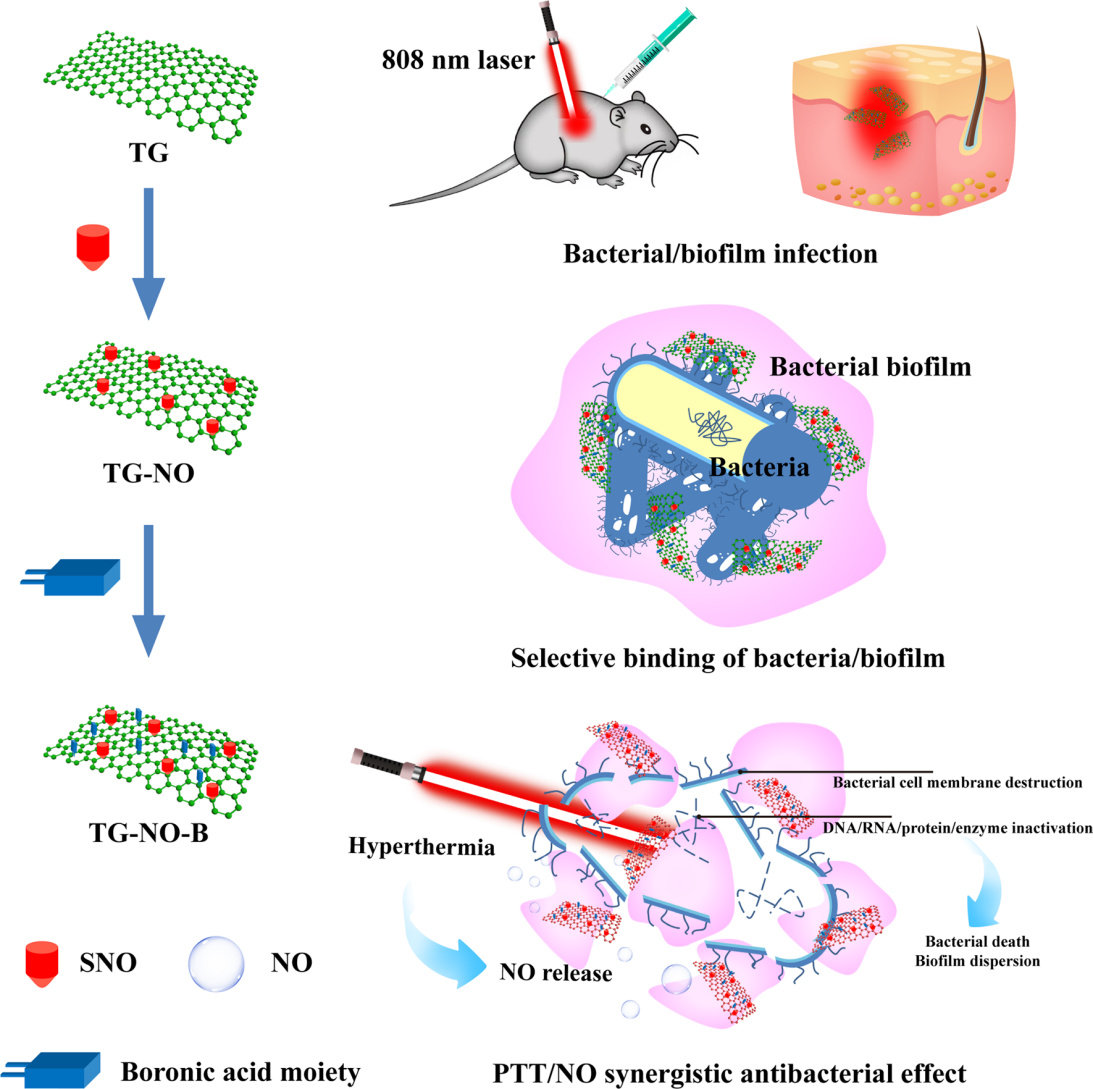
Schematic illustration of the antimicrobial mechanism of TG-NO-B

Based on all the above considerations, we propose a multifunctional platform with single NIR laser-triggered PTT and NO release for synergistic therapy against MDR Gram-negative bacteria and their biofilms. We introduced a kind of photothermal-sensitive NO donor-S-nitrosothiols (SNO) onto thiolated graphene (TG), and then functionalized with 4-mercaptophenylboronic acid (B) to obtain TG-NO-B (Fig. 1 and Additional file 1: Fig. S1). When located at the sites of Gram-negative bacteria-associated infections, TG-NO-B is able to selectively bind to the surfaces of Gram-negative bacterial cells and their biofilm matrix through covalent coupling between the BA groups of TG-NO-B and the bacterial LPS units, which can greatly improve the antibacterial efficiency and reduce undesirable side damages to surrounding healthy tissues. Once exposed to an 808 nm NIR laser, hyperthermia and NO releasing will be induced simultaneously, which can disrupt bacterial cell membrane, further cause leakage and damage of intracellular components, and finally induce bacteria death. On one hand, hyperthermia can induce bacterial cell membrane destruction and DNA/RNA/protein/enzyme inactivation (Fig. 1). On the other hand, hyperthermia can precisely control on-demand release of NO, which interacts with bacteria, and can induce bacterial cell membrane destruction and oxidative/nitrosative stress-oriented DNA damage (Fig. 1). Furthermore, bacterial cell membrane damage can improve the permeability and sensitivity to heat, and decrease the PTT temperature (Fig. 1). We investigated the PTT effect, the NO release behavior, the bacteria-targeting ability and the PTT/NO synergistic antibacterial effect against MDR Gram-negative *Pseudomonas aeruginosa* (*Pa*), *Klebsiella pneumonia* (*Kp*) and *Acinetobacter baumannii* (*Ab*), as well as the synergistic eradication effect on bacterial biofilms in vitro and in vivo (Fig. 1).

## Methods

### Materials

Thiolated graphene (TG) nanosheets were purchased from XFNANO Materials Tech Co., Ltd (Nanjing, China). 2,2′-Dipyridyl disulfide, *tert*-Butyl nitrite (TBN) and 4-mercaptophenylboronic acid were bought from J&K Co., Ltd. (Beijing, China). N, N-Dimethylformamide (DMF) was provided by Sigma-Aldrich Corp. (St. Louis, USA). Cy5-PEG-SH was obtained from Aladdin Reagent Co. Ltd. (Shanghai, China). The LIVE/DEAD BacLight Bacterial Viability Kit was bought from Molecular Probes Invitrogen Corp (Carlsbad, CA, USA). All other chemicals were of analytical grade and used as received unless specially mentioned. Deionized water purified by a Milli-Q water system was applied throughout all experiments.

Three representative multidrug-resistant (MDR) Gram-negative bacterial species, *Acinetobacter baumannii* (*Ab*), *Klebsiella pneumoniae* (*Kp*) and *Pseudomonas aeruginosa* (*Pa*), isolated from human clinical specimens, were obtained from Clinical Microbiology Laboratory, Institute of Burn Research, Southwest Hospital, Army Medical University (AMU, Chongqing, China). Nutrient agar for bacterial culture was provided by Pang Tong Medical Instrument Co. Ltd. (Chongqing, China). NIH 3T3 fibroblast cells were acquired from the Cell Bank of Typical Culture Collection of Chinese Academy of Sciences (Shanghai, China). Dulbecco’s Modified Eagle Medium (DMEM, high glucose) for cell culture was purchased from Gibco Thermo Fisher Scientific Co. Ltd. (Shanghai, China). BALB/C mice (20–25 g, 6–8 weeks) were obtained from Experimental Animal Department of Army Medical University. All the animal experiments followed the ethical principle of Institutional Animal Care and Use Committee of Army Medical University.

### Synthesis of thiolated graphene based nitric oxide nanogenerators (TG-NO)

Firstly, TG nanosheets (2 mg) were dispersed in 4 mL of DMF. Then, *tert*-Butyl nitrite (600 µL) and toluene (2.5 mL) were added into the TG suspension and let stand 24 h under dark conditions at room temperature. Next, the products were centrifuged (10,000 rpm, 60 min) and washed with deionized water and ethanol several times to remove free substrates. The as-prepared compound, as well as 2,2′-dithiodipyridine, was resuspended in 4 mL of ethanol containing 1.8 µL of acetic acid, and then stirred overnight at room temperature to obtain thiolated graphene based NO nanogenerators (TG-NO).

### Synthesis of boronic acid functionalized thiolated graphene based NO nanogenerators (TG-NO-B)

The synthesized TG-NO nanosheets (2 mg) were added into 10 mL of 2-[4-(2-hydroxyethyl)-1-piperazinyl] ethane sulfonic acid (HEPES) solution (pH 7.4) that contained 4-mercaptophenylboric acid (2 mg) and continuously stirred for 18 h at room temperature to obtain TG-NO functionalized with boronic acid (TG-NO-B).

### Cy5-PEG-SH labeling

To synthesize the Cy5-PEG-SH labeled TG-NO-B (Cy5-TG-NO-B), the as prepared TG-NO-B nanosheets (2 mg) were added into 10 mL of 2-[4-(2-hydroxyethyl)-1-piperazinyl] ethane sulfonic acid (HEPES) solution (pH 7.4) that contained Cy5-PEG-SH (1 mg) and continuously stirred for 24 h at room temperature. Subsequently, Cy5-TG-NO-B was obtained by centrifugation at 10,000 rpm for 60 min and dialysis for 48 h. All of these steps were carried out in the dark.

### Characterization

To characterize the chemical structures of TG, TG-NO and TG-NO-B, Fourier transform infrared (FTIR) spectra obtained by the Nicolet 6700 FTIR instrument (Thermo Fisher Scientific Inc., USA) were collected from 32 scans at a resolution of 4/cm between 400 and 4000/cm. X-ray photoelectron spectroscopy (XPS) analysis was performed on an X-ray photoelectron spectrometer (Thermo Fisher ESCALAB 250Xi, USA). The UV–Vis-NIR and fluorescence spectra of Cy5-PEG-SH, TG-NO-B and Cy5-TG-NO-B were analyzed using a spectrophotometer (UV-3600, SHIMADZU, Japan). The sizes and morphologies of TG, TG-NO and TG-NO-B were measured by the dynamic light scattering (DLS) technique (Zetasizer Nano ZEN5600 instrument, Malvern Instruments, UK), transmission electron microscopy (JEOL JEM-1400 TEM analyzer, Japan) and atomic force microscopy (Nano Wizard II AFM system, JPK Instruments, Germany).

### Measurement of optical and photothermal properties

The optical properties of TG, TG-NO and TG-NO-B were characterized by a UV–vis-NIR spectrophotometer (UV-3600, SHIMADZU, Japan). Further, to investigate the photothermal performance of TG-NO-B, TG-NO-B with different concentrations (0–200 μg/mL) was firstly prepared by gradient dilution. Then, each as-prepared suspension (100 µL) was illuminated with an 808 nm NIR laser (power density: 0.25–1.0 W/cm^2^; exposure duration: 0-600 s). The real-time temperature was measured by an infrared camera (FLIR-E49001, Estonia) with an accuracy of 0.1 °C. Finally, the temperature curves were illustrated and the photothermal conversion efficiency (η) was also calculated as previously reported [37].

### Detection of NO release

To investigate the NO release behavior of TG-NO-B, the Griess Reagent Kit (Sigma-Aldrich Corp. St. Louis, USA) was employed. When TG-NO-B was exposed to an 808 nm NIR laser at 0.75 W/cm^2^ for a certain time, the released NO molecules were readily converted into nitrite in aqueous solution and then reacted with the Griess reagent to form a purple azo product, absorbance of which was measured at 546 nm using a microplate reader (Thermo Varioskan Flash, USA).

### In vitro experiments

#### Culture of bacteria and their biofilms

Firstly, a single isolated colony of bacteria on a solid Luria–Bertani (LB) agar plate was transferred to 4 mL of LB broth medium and grown overnight at 37 °C under 230 rpm rotation. The resultant bacteria at the exponential growth phase were harvested and subsequently the bacterial cultures were centrifuged at 6000 rpm for 5 min, and then the bacterial pellets were washed with sterile phosphate buffer solution (PBS) three times to remove medium constituents and other chemical macromolecules. Finally, the pellets were resuspended in PBS, and the optical densities at 600 nm (OD_600_) of bacterial cultures were adjusted to 0.4–0.5 for the following experiments, which corresponded to a concentration of approximately 10^8^ colony-forming units per milliliter (CFU/mL).

The bacteria in culture medium, harvested at the exponential growth phase as mentioned, were transferred to a 96-well plate, kept standing and incubated at 37 °C for 48 h. Then, the suspensions in the plate were removed and the biofilms at the bottom of the plate were obtained and washed with PBS.

#### Assessment of bacteria- and biofilms- targeting abilities in vitro

The abilities of targeting bacteria and their biofilms of TG-NO-B in vitro were assessed by the solution-turbidity method, infrared thermographic technology, scanning electron microscope (SEM) and confocal laser scanning microscope (CLSM). Briefly, the as-prepared bacterial solution (900 μl) was co-incubated with 100 μL of TG-NO-B (1 mg/mL) for 30 min at 37 °C. Next, the value of optical density at 600 nm for the supernatant fluid of the co-incubated solution was measured using a microplate reader (Thermo Varioskan Flash, USA). Then, the suspension was centrifuged at 6000 rpm for 5 min and washed with PBS three times to remove free nanosheets completely. After that, the pellet (TG-NO-B conjugated bacteria) at the bottom was re-suspended in 1 mL of PBS. As-prepared resuspension (100 µL) was irradiated with an 808 nm continuous-wave NIR laser (0.75 W/cm^2^, 600 s). The temperature change was monitored using an infrared camera (FLIR-E49001, Estonia) with an accuracy of 0.1 °C. Further, to observe the morphology of the pellet, it was fixed in 4% (w/v) paraformaldehyde overnight and then dehydrated by a series of ethanol solutions (30%, 50%, 70%, 95% and 100%). Subsequently, the sample was dried in a vacuum drying chamber. After gold sputtering, the sample was observed using SEM (Crossbeam 340, Zeiss, Germany). Control experiments were conducted in parallel with PBS, TG, TG-B and TG-NO, respectively.

The in vitro interactions between TG-NO-B or its control counterparts and the bacterial biofilms were also investigated using the same methods (the SEM observation and photothermal-induced temperature changes).

To explore whether TG-NO-B could interact with the host cells, the test nanosheets (TG, TG-NO, TG-B, TG-NO-B) were co-incubated with 3T3 fibroblast cells for 24 h. Then, the cells were washed with sterile PBS three times to remove free nanosheets. The SEM observation and measurement of temperature change (under the 808 nm NIR laser, 0.75 W/cm^2^, 10 min) were conducted as described previously.

#### Evaluation of antibacterial activity in vitro

To evaluate the antibacterial activity of TG-NO-B in vitro, the standard plate counting method and Live/Dead staining assay were used to determine the bacterial viability. Firstly, 900 μL of the bacterial solution was mixed with 100 μL of PBS or the as-prepared nanosheets (TG, TG-NO, TG-B or TG-NO-B, 1 mg/mL), and co-incubated for 4 h at 37 °C with constant shaking. Then, the NIR treated groups were further irradiated with an 808 nm laser (0.75 W/cm^2^) for another 10 min.

For standard plate counting assay, the resultant bacterial solution was sequentially diluted and uniformly spread onto the agar plates, followed by incubation at 37 °C for 18 h. The images and counting of bacterial colonies were obtained using an automatic colony counter (Supcre, Shineso, Hangzhou).

For Live/Dead staining assay, the bacteria were harvested by centrifugation at 6000 rpm for 5 min and stained by the Live/Dead staining kit (Invitrogen, USA) for 30 min in dark according to the instruction manual. Afterwards, the staining bacteria were washed with sterile PBS twice and observed under a fluorescence microscopy (Zeiss LSM780, Germany). The loss of bacterial viability was calculated as the number of dead cells (in red)/the number of total cells (in green) × 100%.

The morphological change of bacterial membrane after the TG-NO-B + NIR treatment was examined by SEM measurement. The resultant bacteria were harvested by centrifugation at 6000 rpm. Next, the collected bacteria were prepared for SEM observation as previously described. Bacterial cell membrane integrity was further determined by detecting the leakage of intracellular DNA/RNA. Briefly, 100 μL of the test nanosheets (TG, TG-NO, TG-B, TG-NO-B, 100 μg/mL) were co-incubated with 900 μL of bacterial solution for 4 h at 37 °C with constant shaking. Then, the resultant bacterial suspensions were further exposed to an 808 nm laser (0.75 W/cm^2^, 600 s). After filtration with 0.2-μm syringe filters to remove bacteria and nanomaterials, the efflux of cytoplasmic nucleic acids from the bacteria into the supernatant was measured with a microplate reader (Thermo Varioskan Flash, USA) at 260 nm.

#### Evaluation of biofilm dispersion ability in vitro

The in vitro biofilm dispersion ability of TG-NO-B was evaluated by crystal violet (CV) staining assay and standard plate counting assay. Firstly, different concentrations of TG-NO-B were co-incubated with the as-prepared biofilms, followed by incubation at 37 °C for 6 h. The resultant biofilms were rinsed with PBS three times and irradiated with a NIR laser (808 nm, 0.75 W/cm^2^) for 10 min. Next, the biofilms were fixed with 100% (v/v) methanol and then stained with 100 µL of 0.5% (v/v) crystal violet solution. Following 30 min treatment, the samples were vigorously rinsed with sterile PBS three times to remove free dye. After that, 200 µL of 95% (v/v) ethanol was added to dissolve the dye. Finally, the absorbance at 590 nm of the resultant solution was determined by a microplate reader (Thermo Varioskan Flash, USA). Control experiments were performed in parallel with TG, TG-NO and TG-B, respectively. Further, the standard plate counting method was also employed to quantify biofilm bacteria as described earlier.

#### Evaluation of biocompatibility in vitro

##### In vitro cytotoxicity assessment

The in vitro cytotoxicity of TG-NO-B was assessed by CCK-8 (Dojindo, Japan) assay. Firstly, 3T3 fibroblast cells were cultured in 96-well flat bottom plates (3000 cells per well), and incubated for 24 h in a 5% CO_2_ incubator at 37 °C. Afterwards, the cell culture medium was replaced with fresh medium containing various concentrations of TG-NO-B (50, 100 and 500 µg/mL). After incubation for the indicated time periods, CCK-8 solution was added into each test well according to the manufacturer’s instruction. Following further incubation for another 3 h at 37 °C, the absorbance at 450 nm was measured using a microplate reader (Thermo Varioskan Flash, USA).

##### In vitro hemolysis assay

The hemolytic activity was studied using fresh human blood samples, acquired from Southwest Hospital with patients’ written informed consent. Firstly, the whole blood sample was centrifuged at 1500 rpm for 15 min and rinsed three times with saline [0.9% (w/v) NaCl] to collect erythrocytes. Subsequently, the stock dispersion was prepared by mixing 3 mL of centrifuged erythrocytes with 11 mL of saline. Next, 100 µL of the stock dispersion was respectively added into 1 mL of various concentrations of TG-NO-B (50, 100 and 500 µg/mL). The saline and deionized water were set as negative control and positive control, respectively. After incubation at 37 °C for 3 h, the resultant solution was centrifuged at 12,000 rpm for 15 min, and the absorbance at 540 nm of the supernatant was determined using a microplate reader (Thermo Varioskan Flash, USA). The percentage of hemolysis was calculated according to the following formula:

$${\text{Hemolysis}}\;\left( \% \right) = {{\left( {{\text{AE}} - {\text{AN}}} \right)} \mathord{\left/ {\vphantom {{\left( {{\text{AE}} - {\text{AN}}} \right)} {{\text{AP}} - {\text{AN}}}}} \right. \kern-0pt} {{\text{AP}} - {\text{AN}}}} \times 100\% .$$

where AE was the absorbance of the experimental group, AN was the absorbance of the negative control, and AP was the absorbance of the positive control.

### In vivo experiments

#### Animal model

For in vivo experiments, the animal model of murine-infected full-thickness skin defect wound and subcutaneous abscess were developed. BALB/c mice (18-20 g, 6–8 weeks) were anaesthetized with 1% (w/v) pentobarbital via intraperitoneal injection (0.01 mg/g of body weight). Firstly, the dorsal hair of each mouse was shaved and the exposed skin was sterilized with 75% (v/v) ethanol. Afterwards, for the murine-infected full-thickness skin defect wound model, one circular full-thickness wound with 6-mm diameter was created with a punch on each side of the back. The skin wound was inoculated with a 5 μL aliquot of bacterial suspension (OD_600_ = 0.5). After 24 h treatment, the wound was found to be infected. For the subcutaneous abscess model, 50 μL aliquot of bacterial suspension (OD_600_ = 0.5) was injected into the subcutaneous tissue on the back of test mouse. After injection for 24 h, a subcutaneous abscess successfully formed at the injected site.

#### Evaluation of bacteria- and biofilms-targeting ability in vivo

To verify the in vivo bacteria- and biofilms-targeting ability of TG-NO-B, the infected site of the test mouse (infected wound or subcutaneous abscess) was injected with 50 μL of TG-NO-B (100 μg/mL). As a control, the same amount of TG-NO-B was injected into the uninfected site (clean wound or normal skin) on the other flank of the same mouse. Then, each side of the back of the test mouse was separately illuminated with an 808 nm NIR laser (0.75 W/cm^2^, 10 min) at the predetermined time points after injection (0, 2, 4, 6 and 24 h). The thermographic images were taken by an IR thermal camera (FLIR-E49001, Estonia).

To further explore the biodistribution of TG-NO-B via intravenous injection, the mouse model was administered with Cy5-TG-NO-B (150 μL, 100 μg/mL) via intravenous injection. NIRF images were recorded as previously described. At the indicated time points (days 1, 4, 7, 10) after postinjection, the test mice were sacrificed. The major organs (heart, liver, spleen, lung and kidney) and infected tissues were harvested and imaged by the IVIS Lumina II imaging system.

#### In vivo antimicrobial and anti-biofilm therapy

The murine infected full-thickness skin defect wound model and subcutaneous abscess model were utilized to investigate the in vivo antibacterial activity and anti-biofilm activity, respectively. The test mice were randomly divided into 5 groups (n = 5 in each group): PBS, TG, TG-NO, TG-B and TG-NO-B.

For in vivo antibacterial therapy, each infected wound was treated with 100 μL of TG, TG-NO, TG-B or TG-NO-B dispersions at 100 μg/mL or PBS (control group), respectively. Blank group without bacterial inoculation was used as the negative control. At 6 h post-inoculation, the wound was exposed to an 808 nm NIR laser (0.75 W/cm^2^) for 10 min. The temperature of the wound was monitored using an IR thermal camera (FLIR-E49001, Estonia). At day 1, 3, 5, and 7 post-treatment, the wound was photographed. A 6-mm-diameter sterile round marker was placed beside each wound to represent the initial wound area. The wound area was measured by Image Pro Plus 6.0 software (Media Cybernetics, Silver Spring, USA). The wound healing rate was calculated using the following formula:

$${\text{Wound}}\;{\text{healing}}\;{\text{rate}}\;(\% ) = {{\left( {{\text{I}} - {\text{R}}} \right)} \mathord{\left/ {\vphantom {{\left( {{\text{I}} - {\text{R}}} \right)} {\text{I}}}} \right. \kern-0pt} {\text{I}}} \times 100\% .$$

where I represents the initial wound area, and R represents the residual wound area on the determined day post-treatment.

At the indicated time points, the test mouse was sacrificed, and the infected tissues were collected for bacterial colony counting (standard plate counting method) and histological analysis (HE staining).

Similarly, PBS, TG, TG-NO, TG-B and TG-NO-B was respectively injected into the subcutaneous abscess of the test mice for anti-biofilm therapy in vivo. After 6 h post-injection, the subcutaneous abscess was irradiated with an 808 nm NIR laser (0.75 W/cm^2^) for 10 min. The real-time temperature of the abscess was measured with an IR thermal camera (FLIR-E49001, Estonia). At day 12 post-treatment, the abscess was biopsied and photographed. At the same time, the infected tissues were harvested for further bacterial colony counting and histological analysis.

#### In vivo toxicity evaluation

BALB/c mice (20–25 g, 6–8 weeks, 5 mice per group) were used to investigate the in vivo toxicity of TG-NO-B. After intravenous injection of 100 μL of sterile PBS or TG-NO-B dispersions (50, 100 and 500 μg/mL), the mice were sacrificed at the predetermined times post-injection (7 or 28 days). The major organs (heart, liver, kidney, spleen and lung) were biopsied and washed with PBS. Then, the organs were fixed with 4% (w/v) paraformaldehyde, embedded in paraffin, sectioned at a thickness of 5 μm and stained with hematoxylin and eosin (H&E). Histological examination was carried out under an optical microscope (CTR600, Leica, Germany). Moreover, blood samples of the test mice were also collected and analyzed by blood cell count and serum biochemistry.

### Statistical analysis

The experimental data are expressed as mean ± standard deviation (SD), and the significant difference between groups was analyzed using unpaired t test (for two groups) and one-way analysis of variance (ANOVA) (for more than two groups) with IBM SPSS statistics 23.0. The statistical significance was set as *p* < 0.05 (“*”) and *p* < 0.01 (“**”), respectively.

## Results and discussion

### Fabrication and characterization of TG-NO-B

**Fig. 2:**
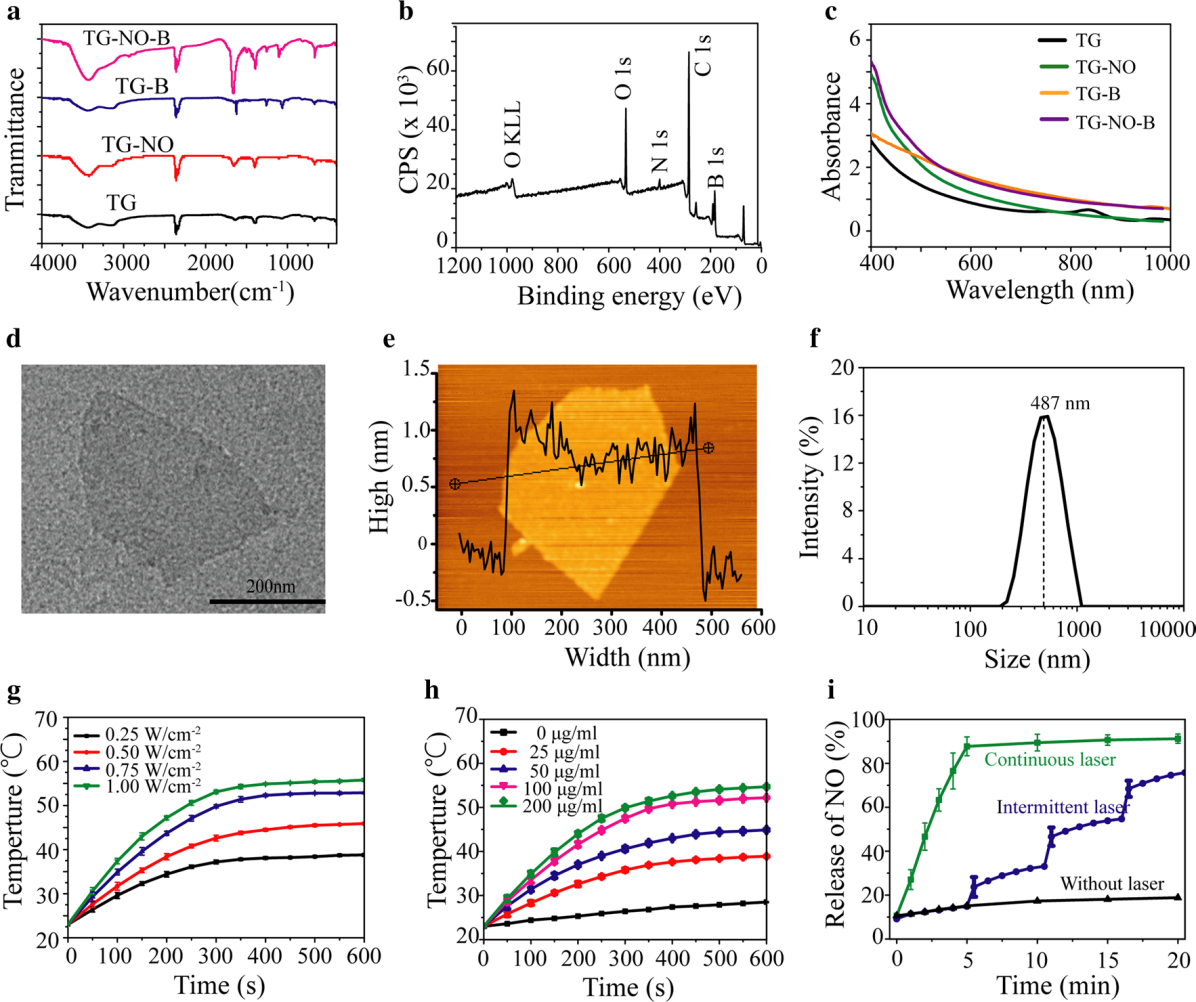
**a** FTIR-ATR spectra of synthesized TG, TG-NO, TG-B and TG-NO-B. **b** XPS survey of TG-NO-B. **c** UV–Vis-NIR spectra of TG, TG-NO, TG-B and TG-NO-B. **d**TEM and **e** AFM images of TG-NO-B nanosheets. **f** Hydrodynamic diameter of TG-NO-B in PBS buffer measured by DLS. **g** Temperature evolution curves of TG-NO-B (100 μg/mL) under 808 nm NIR irradiation at different power densities (0.25–1.0 W/cm^2^). **h** Temperature evolution curves of TG-NO-B with different concentrations (0-200 μg/mL) under 808 nm NIR irradiation at 0.75 W/cm^2^. **i** NO release profile of TG-NO-B under different laser irradiation conditions

The fabrication of the TG-NO-B was illustrated in Additional file 1: Fig. S1. Firstly, S-nitrosothiols (SNO) were conjugated to the thiolated graphene (TG) nanosheets by reacting the -SH groups with tert-butyl nitrite (TBN) to form the TG-NO, as previously reported [38]. Afterward, the boronic acid (BA) groups were linked to the surfaces of TG-NO to obtain the final TG-NO-B. Simultaneously, the TG-B nanosheets were also synthesized by grafting the BA groups onto the surfaces of TG. After purification, the obtained TG-NO, TG-B and TG-NO-B were characterized by using the FTIR and XPS analysis. As presented in Fig. 2a, the FTIR spectrum of TG-NO-B revealed a peak of 1385/cm, the character of S–N, and a peak of 1095/cm, which indicates the appearance of S–S. The above two peaks were also observed in the spectra of TG-NO and TG-B, respectively. Furthermore, the XPS analysis of TG-NO-B revealed the coexistence of the nitrogen (N) and boron (B) elements (Fig. 2b). These results together confirmed the successful synthesis of TG-NO, TG-B and TG-NO-B.

The morphological features and size distributions of the as-prepared TG-NO, TG-B and TG-NO-B nanosheets were investigated by using TEM, AFM and DLS. TEM characterization suggested that the functionalization with SNO and BA caused no obvious changes in the surface features of TG-NO (Additional file 1: Fig. S2D), TG-B (Additional file 1: Fig. S2G) and TG-NO-B (Fig. 2d) compared with TG (Additional file 1: Fig. S2A). Similarly, AFM analysis also revealed that there were no significant differences in the thicknesses of TG-NO (Additional file 1: Fig. S2E), TG-B (Additional file 1: Fig. S2G) and TG-NO-B (Fig. 2e) in comparison with TG (~ 1.5 nm) (Additional file 1: Fig. S2B). Moreover, the hydrodynamic sizes of TG-NO (peak: 429 nm, Additional file 1: Fig. S2F), TG-B (peak: 458 nm, Additional file 1: Fig. S2I) and TG-NO-B (peak: 487 nm, Fig. 2f) all slightly increased as compared to TG (peak: 412 nm, Additional file 1: Fig. S2C). These results suggested that the surface functionalization had no notable influence on the morphological feature and size distribution of graphene nanosheets, and the TG-NO, TG-B and TG-NO-B nanosheets were still nearly single-layered and uniformly dispersed.

### Optical and photothermal properties of TG-NO-B

Graphene and its derivatives have been considered as excellent photothermal agents owing to their good photostability, high absorption in the UV-NIR region and strong light-induced heating effects [16, 39]. An 808 nm NIR laser as the optimal light source is widely used for graphene-based photothermal therapy (PTT) due to its deep penetration of skin and low absorbance by tissue [9, 40]. Thus, the optical absorption of TG, TG-NO, TG-B and TG-NO-B was first measured using a UV–Vis-NIR spectrometer. As shown in Fig. 2c, TG, TG-NO, TG-B and TG-NO-B exhibited an absorption over a broad range in the Vis–NIR region with the absorbances of 0.604, 0.528, 0.977 and 0.928 at 808 nm, respectively. Both TG-B and TG-NO-B showed a higher absorption at 808 nm, probably because of the enhanced electron transfer by grafting BA moieties onto the graphene sheets [41, 42]. Next, the photothermal effect of TG-NO-B was investigated by measuring the temperature evolution profile of its suspensions (0–200 μg/mL) under 808 nm laser irradiation (0.25–1.0 W/cm^2^, 600 s). As shown in Fig. 2g, the temperatures of 100 μg/mL TG-NO-B rose with the increase of the laser power intensity, and all reached a plateau within 10 min. Moreover, the temperatures of TG-NO-B were also rapidly elevated in a concentration-dependent manner upon the laser irradiation at 0.75 W/cm^2^ (Fig. 2h). In contrast, nearly no big temperature change was observed for PBS (0 μg/mL, 0.75 W/cm^2^) (Fig. 2h). Such results demonstrated that TG-NO-B could effectively transform infrared light energy into heat energy. Moreover, the photothermal conversion efficiency (η) was calculated according to previously reported method [37]. As depicted in Additional file 1: Fig. S3, TG-NO-B exhibited a high η value of 37.6%, making it very excellent as a promising PTT agent. Furthermore, irreversible thermal ablation of bacteria always occurs at the temperature over 55 °C, but the damage of bacterial cell membranes may enhance permeability and sensitivity to heat, consequently making bacteria more vulnerable and requiring lower photothermal temperatures [20, 43]. 100 μg/mL TG-NO-B under the laser irradiation at 0.75 W/cm^2^ for 10 min possessed an equilibrium temperature of 49.8 °C, and was selected to explore the following chemo-photothermal synergistic effect against bacteria, considering the destructive effect of NO on bacterial membranes.

### Controllable NO release of TG-NO-B

To investigate the NO generation property of TG-NO-B, the release of NO in aqueous solution was quantitatively measured using the Griess assay. The NO release profiles of different groups were shown in Fig. 2i. For TG-NO-B without laser irradiation, NO was released in a slow and steady manner with only approximately 18.8% of the total NO releasing in 20 min. By contrast, when being treated with continuous laser irradiation (0.75 W/cm^2^), TG-NO-B revealed a burst NO release within 5 min with almost 87.7% of the total NO releasing. These results suggest that laser irradiation can boost NO release from TG-NO-B, most probably ascribed to the breakdown of S-NO bonds induced by the heat energy from laser irradiation [38, 44]. Notably, a controllable NO releasing pattern was observed under intermittent laser irradiation (30 s per 5 min). Specifically, a steep increase of NO production appeared when the laser irradiation was on for 30 s. While the laser irradiation was switched off, the NO releasing was greatly attenuated. The above results suggest that the as-prepared TG-NO-B can achieve NIR triggered-controllable NO release, making it a potential candidate for biomedical applications.

### In vitro bacteria- and biofilms-targeting abilities of TG-NO-B

**Fig. 3:**
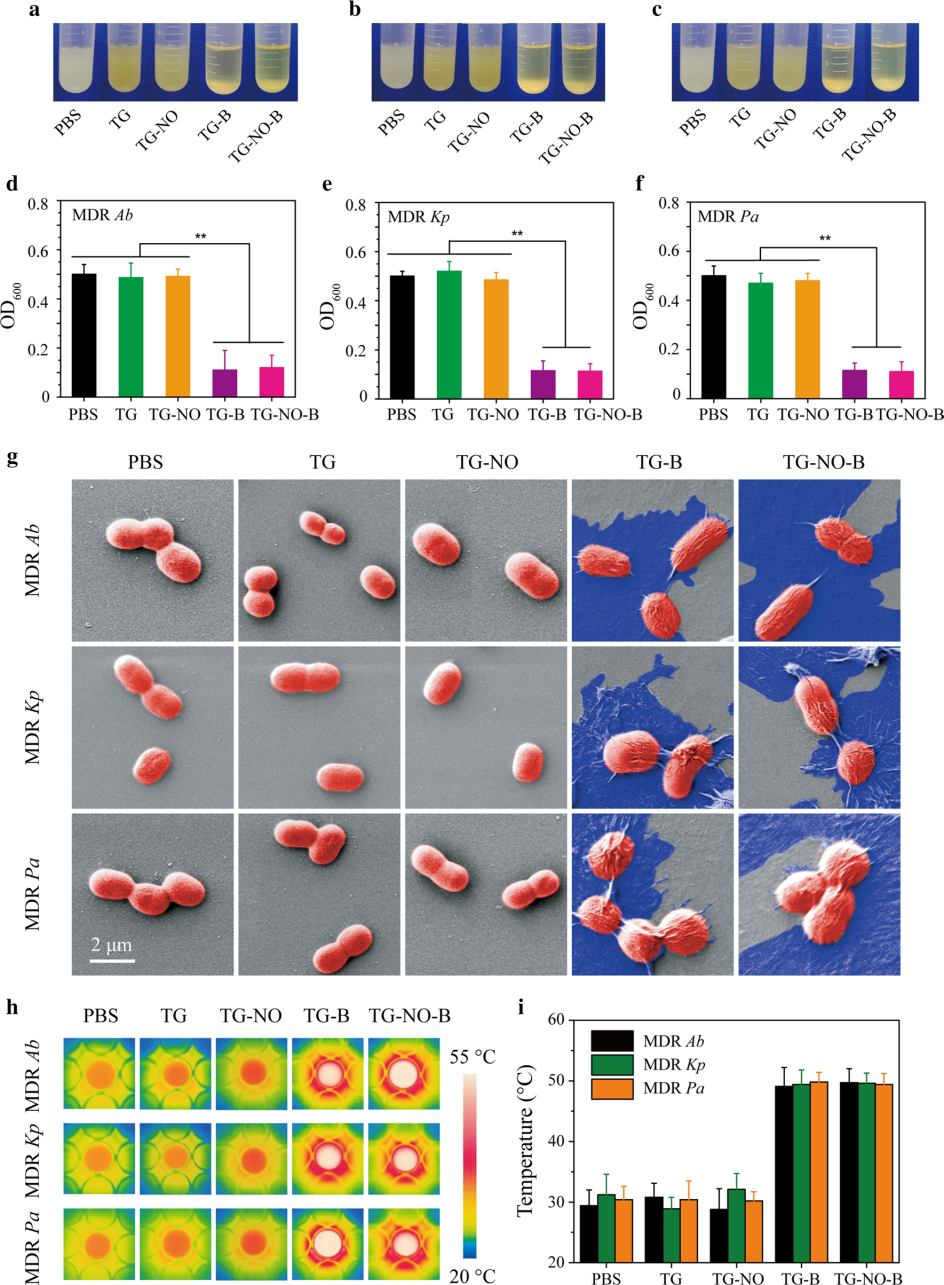
Photographs of bacterial suspensions of **a** MDR *Ab*, **b** MDR *Kp* and **d** MDR *Kp* after incubation with different nanomaterials. The corresponding OD_600_ values of the supernatants for **d** MDR *Ab*, **e** MDR *Kp* and **f** MDR *Kp* suspensions after incubation with different nanomaterials. **g** Representative SEM images of MDR *Ab*, MDR *Kp* and MDR *Pa* after incubation with PBS, TG, TG-NO, TG-B and TG-NO-B. **h** Temperature measurements and **i** the corresponding thermographic images of MDR *Ab*, MDR *Kp* and MDR *Pa* suspensions after incubation with PBS, TG, TG-NO, TG-B and TG-NO-B under NIR irradiation (10 min, 0.75 W/cm^2^)

The cell walls and biofilm matrix of Gram-negative bacteria are high in polysaccharides [45, 46], which are able to be covalently bound by boronic acids (BA) to form cyclic boronic esters [35]. Therefore, we hypothesize that TG-NO-B should be capable of selectively binding to Gram-negative bacteria or their biofilms through the strong covalent interaction between the BA moieties of TG-NO-B and the diol groups of bacterial polysaccharides while minimizing the direct interaction with host cells. To verify our hypothesis, MDR Gram-negative bacteria -*Ab, Kp* and *Pa* were chosen as the model bacteria and co-incubated with TG, TG-NO, TG-B and TG-NO-B. Firstly, the bacterial binding property of TG-NO-B was investigated by a turbidity method. As presented in Fig. 3a, precipitates were clearly observed at the bottom of the test tubes in the TG-B- and TG-NO-B-treated groups within 30 min, owing to the conjugation of TG-B/TG-NO-B and MDR *Ab*. The turbidity measurements showed that the OD_600_ values of the corresponding supernatants decreased to 0.11 ± 0.08 and 0.13 ± 0.05, respectively (Fig. 3d). On the contrary, precipitation phenomena were not found in PBS-, TG- and TG-NO-treated groups (Fig. 3a), and the OD_600_ values of the corresponding supernatants were 0.50 ± 0.04,0.48 ± 0.06 and 0.49 ± 0.03, respectively (Fig. 3d). Similar results were observed for MDR *Kp* (Fig. 3b, e) and MDR *Pa* (Fig. 3c, f) as well. Next, after that the free nanomaterials were removed by centrifugation and rinsing, SEM was further utilized to visually confirm the binding ability of TG-NO-B towards the bacteria. As shown in Fig. 3g, the bacterial cells (red) were firmly encapsulated by the TG-B and TG-NO-B nanosheets (blue), while no TG or TG-NO nanosheets were bound to the bacterial surfaces. Moreover, the specific interaction between the bacteria and TG-NO-B was demonstrated by thermographic imaging. Following removing the free nanosheets completely, the resultants were exposed to an NIR laser (808 nm, 0.75 W/cm^2^) for 10 min. As shown in Fig. 3h, i, the temperatures in the PBS-, TG- and TG-NO-treated groups all only kept around 30 °C. In contrast, the temperatures in the TG-B/TG-NO-B-treated groups reached 49.1 ± 3.1/49.7 ± 2.3 °C (for MDR *Ab*), 49.4 ± 2.4/49.6 ± 1.7 °C (for MDR *Kp*) and 49.8 ± 1.6/49.4 ± 1.8 °C (for MDR *Pa*), respectively. The increased temperatures of the TG-B- and TG-NO-B-treated groups could be attributed to their high affinities towards bacteria.

To further certificate the possibility of the adherence and aggregation of TG-NO-B nanosheets on the bacterial biofilms, SEM analysis and thermographic imaging were also performed as before. The results were similar to our findings on the planktonic bacteria. As presented in Additional file 1: Fig. S4A, a great number of TG-B and TG-NO-B nanosheets tightly attached to the bacterial biofilms; while nearly no TG and TG-NO nanosheets were found on the biofilms. Moreover, upon NIR laser irradiation (808 nm, 0.75 W/cm^2^, 10 min), the temperatures of the TG-B- and TG-NO-B-treated groups all reached as high as approximately 50 °C; whereas the temperatures of the PBS-, TG- and TG-NO-treated groups were about 30 °C, 31 °C and 30 °C, respectively.

In addition, NIH/3T3 fibroblasts were used as the model to explore the possible interaction between the nanomaterials and host cells. Briefly, the test cells were cultured in the mediums containing TG-NO-B and the control counterparts (PBS, TG, TG-NO and TG-B) for 24 h. Next, the cells were repeatedly rinsed, and followed by SEM observation and exposure to an 808 nm laser irradiation (0.75 W/cm^2^, 10 min). Finally, the cell viabilities and temperatures were respectively measured. As shown in Additional file 1: Fig. S5A, the test nanomaterials could not adhere to the cell surfaces, and no obvious morphological changes of the cells were found. Simultaneously, the temperatures did not show significant changes in the TG-NO-B-treated group compared to the control groups (Additional file 1: Fig. S5B, C). The above results reveal that TG-NO-B can be used to achieve specific targeting on MDR Gram-negative bacteria and their biofilms over mammalian cells.

### In vitro antibacterial and anti-biofilm activities of TG-NO-B

**Fig. 4:**
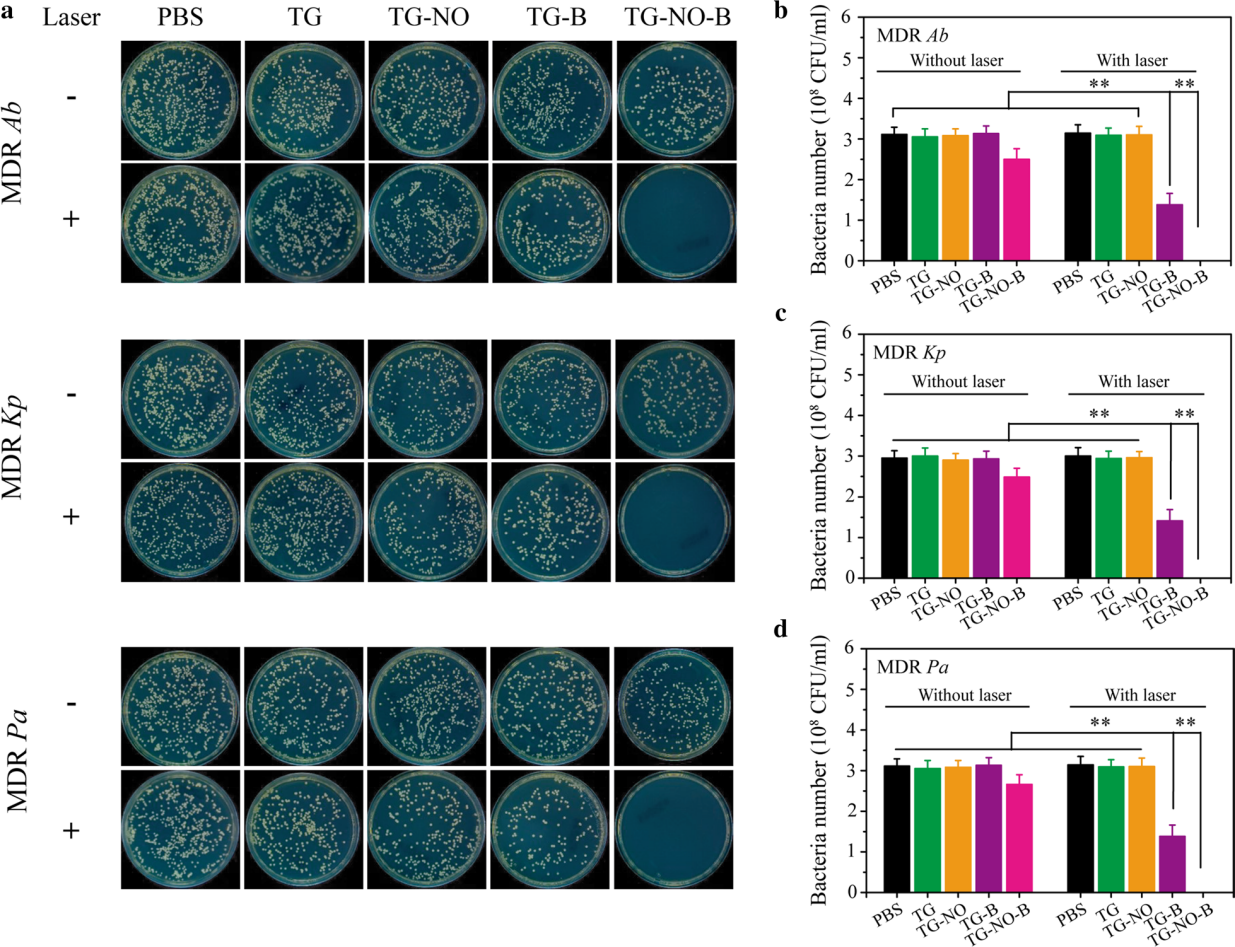
**a** Representative images of bacterial CFUs of MDR *Ab*, MDR *Kp* and MDR *Pa* exposed to PBS, TG, TG-NO, TG-B, TG-NO-B, PBS + NIR, TG +NIR, TG-NO +NIR, TG-B +NIR and TG-NO-B. Quantitative analysis of **b** MDR *Ab*, **c** MDR *Kp* and **d** MDR *Pa* receiving the treatments of PBS, TG, TG-NO, TG-B and TG-NO-B with or without NIR laser irradiation

Nitric oxide (NO), as a typical lipophilic biological signal molecule, is a broad-spectrum antibacterial candidate based on its reducing power and its byproducts peroxynitrite and dinitrogen trioxide, resulting in lipid peroxidation, rupture of cell membranes, DNA cleavage and protein dysfunction [15, 22]. Upon 808 nm NIR laser irradiation, graphene and its derivatives can transfer light energy into heat energy, release it, and eradicate the bacteria and their biofilms [9, 16]. Thus, following successful verification of the photothermal effect and controllable NO release of TG-NO-B, the in vitro antibacterial and anti-biofilm activities were investigated. Figure 4 indicate the plates and colony counts of TG-NO-B and its control counterparts (PBS, TG, TG-NO and TG-B) against three MDR Gram-negative bacterial strains (*Ab, Kp* and *Pa*) with either NIR laser irradiation or not, respectively. Without the laser irradiation, the bacterial survival rates in the TG-NO-B-treated groups were 80.4 ± 8.3%, 84.1 ± 7.5%, and 85.5 ± 7.7% for MDR *Ab*, *Kp* and *Pa*, respectively, compared to the untreated controls (PBS); however, all the other groups without NIR laser irradiation (TG, TG-NO and TG-B) showed no significant changes in the percentages of survival bacteria. The slight bactericidal effects of the TG-NO-B-treated groups could be attributed to targeted and relatively small amount of NO release by TG-NO-B without NIR light [22, 47]. Upon exposure to NIR laser irradiation (808 nm, 0.75 W/cm^2^, 10 min), the TG- and TG-NO-treated groups exhibited no or very little decrease in the bacterial viabilities, due largely to no or weak binding between TG/TG-NO and the bacteria [9]; whereas the inactivated percentages in the TG-B-treated groups were 55.6 ± 9.0%, 55.2 ± 9.5 and 55.6 ± 9.0 for *Ab*, *Kp* and *Pa*, respectively, owing to the occurrence of targeted PTT [35]. Furthermore, TG-B based PTT showed a power-dependent bactericidal activity, but the bacterial survival rates still remained at ~ 35% even at a high laser power density of 1.0 W/cm^2^ (Additional file 1: Fig. S6A). Notably, following NO-based chemotherapy was introduced, TG-NO-B based synergistic chemo-PTT showed the highest antibacterial efficiency as high as 100%. Moreover, as shown in Additional file 1: Fig. S6B and C, the bacteria inactivation effects of TG-NO-B based chemo-PTT followed a power- and dose-dependent manner. At a concentration of 100 μg/mL of TG-NO-B and a laser power density of 0.75 W/cm^2^, all the test bacteria could be completely eliminated. Collectively, compared to the monotherapy of chemo or photothermal, the synergistic chemo-PTT of TG-NO-B exhibited a superior antibacterial activity against MDR Gram-negative bacteria.

**Fig. 5:**
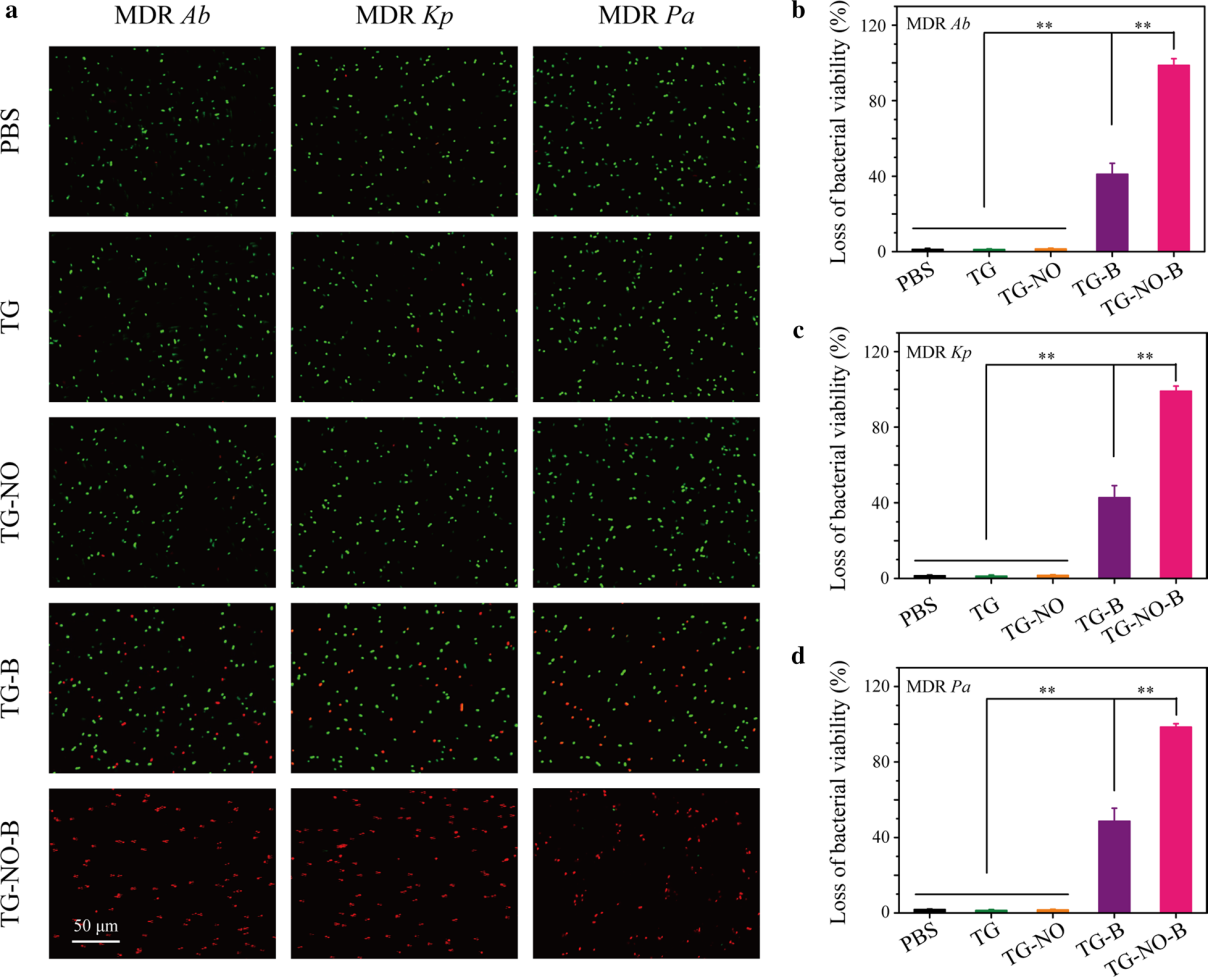
**a** Fluorescence microscope images of MDR *Ab*, MDR *Kp* and MDR *Pa* in PBS, TG, TG-NO, TG-B and TG-NO-B group after being stained with SYTO 9 and PI. Loss of bacteria viability for **b** MDR *Ab*, **c** MDR *Kp* and **d** MDR *Pa* after incubation with PBS, TG, TG-NO, TG-B and TG-NO-B

To further elucidate the antibacterial behavior of the synergistic chemo-PTT of TG-NO-B, a Live/Dead staining assay was performed. All bacteria can be stained with a fluorescent green color by SYTO9, while dead or damaged bacteria with destructive cell membranes can be stained with a fluorescent red color by PI. As shown in Fig. 5a, almost all the bacteria in the PBS/TG + NIR-treated groups were stained as green, while only a few red spots were observed in the TG-NO + NIR treated groups. Furthermore, an increasing number of red fluorescent signals were observed for the TG-B + NIR- and TG-NO-B + NIR-treated groups. The corresponding quantitative analysis of red/green fluorescence was presented in Fig. 5b–d. Compared with the PBS/TG/TG-NO + NIR-treated groups, the TG-B + NIR-treated groups showed a significant reduction in bacterial survival rates (59.0 ± 4.1%, 57.4 ± 3.7% and 51.4 ± 4.6% for MDR *Ab*, *Kp* and *Pa*, respectively; *P* < 0.01), suggesting that the targeted hyperthermia (~ 50 °C) could destruct the cell membrane integrity of some bacteria [48, 49]. More importantly, the bacteria treated with TG-NO-B + NIR exhibited the most dramatic loss of viability (98.7 ± 1.1%, 99.1 ± 0.7% and 98.5 ± 0.5% for MDR *Ab*, *Kp* and *Pa*, respectively; *P* < 0.01), indicating that the synergistic chemo-PTT of TG-NO-B could enhance bactericidal effect highly at a lower photothermal temperature of approximately 50 °C. Consequently, the above results further confirmed that TG-NO-B was a superior chemo-photothermal synergistic system with outstanding antibacterial efficiency.

In addition, the bacterial membrane damage was also confirmed by SEM (Additional file 1: Fig. S7). The untreated bacteria in the control groups had smooth appearances with intact cellular membranes; however, following the treatment with TG-NO-B + NIR, the cytoplasmic membranes wrinkled and the whole cells collapsed. Furthermore, membrane damage of bacteria can lead to the leakage of intracellular components (e.g. DNAs, RNAs, etc.) out of the cytoplasm, further resulting in cell dysfunction and eventually cell death [50]. Since DNAs or RNAs has a characteristic absorption at 260 nm, the leakage of intracellular DNAs and RNAs from the bacterial cells with different treatments was investigated by measuring the OD value at 260 nm (OD_260_) of the culture media. As shown in Additional file 1: Fig. S8, the TG-NO-B + NIR-treated groups exhibited the highest OD_260_ values (0.29 ± 0.03, 0.23 ± 0.05 and 0.26 ± 0.05 for MDR *Ab*, *Kp* and *Pa*, respectively), supporting the strongest destruction of the bacterial membrane. Moreover, a dose-dependent increase of OD_260_ values were observed as the concentration of TG-NO-B ascended. Together, the probable antibacterial mechanism of TG-NO-B is that TG-NO-B specially adsorb onto the bacterial cell membrane, then disrupt the integrity of the membrane via the local hyperthermia and the released NO under NIR laser irradiation, finally lead to leakage of intracellular components like DNAs/RNAs and induce bacteria death [13, 22]. Furthermore, the bacterial cell damage can increase the permeability and sensitivity of bacterial cells to heat and decrease the hyperthermia temperature [20, 43]. On the other hand, the hyperthermia caused by PTT can promote NO release (Fig. 2I), which may further damage the bacterial cells.

**Fig. 6:**
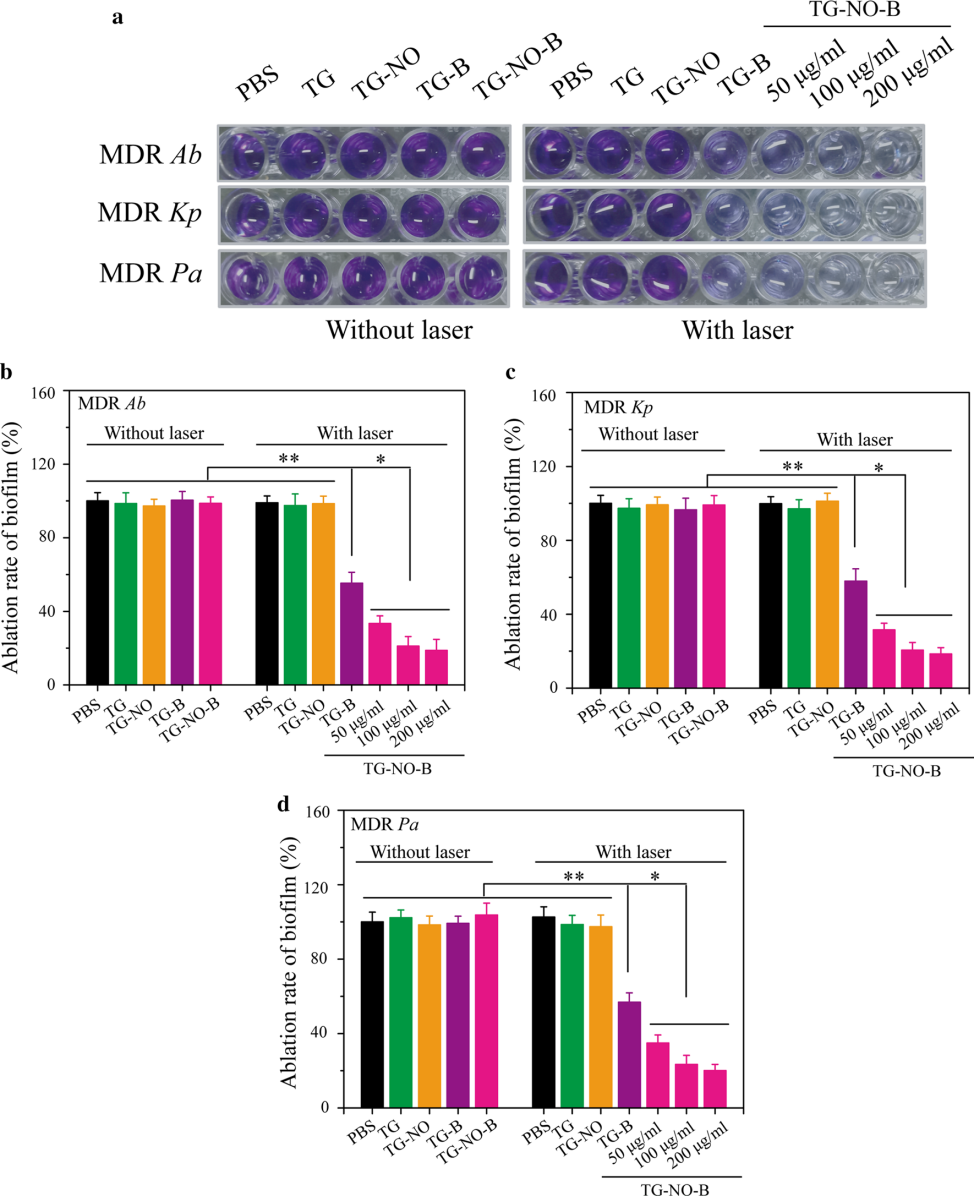
**a** Biofilm stained with crystal violet. Ablation rate of **b** MDR *Ab*, **c** MDR *Kp* and **d** MDR *Pa* biofilm determined by absorbance of crystal violet

**Fig. 7:**
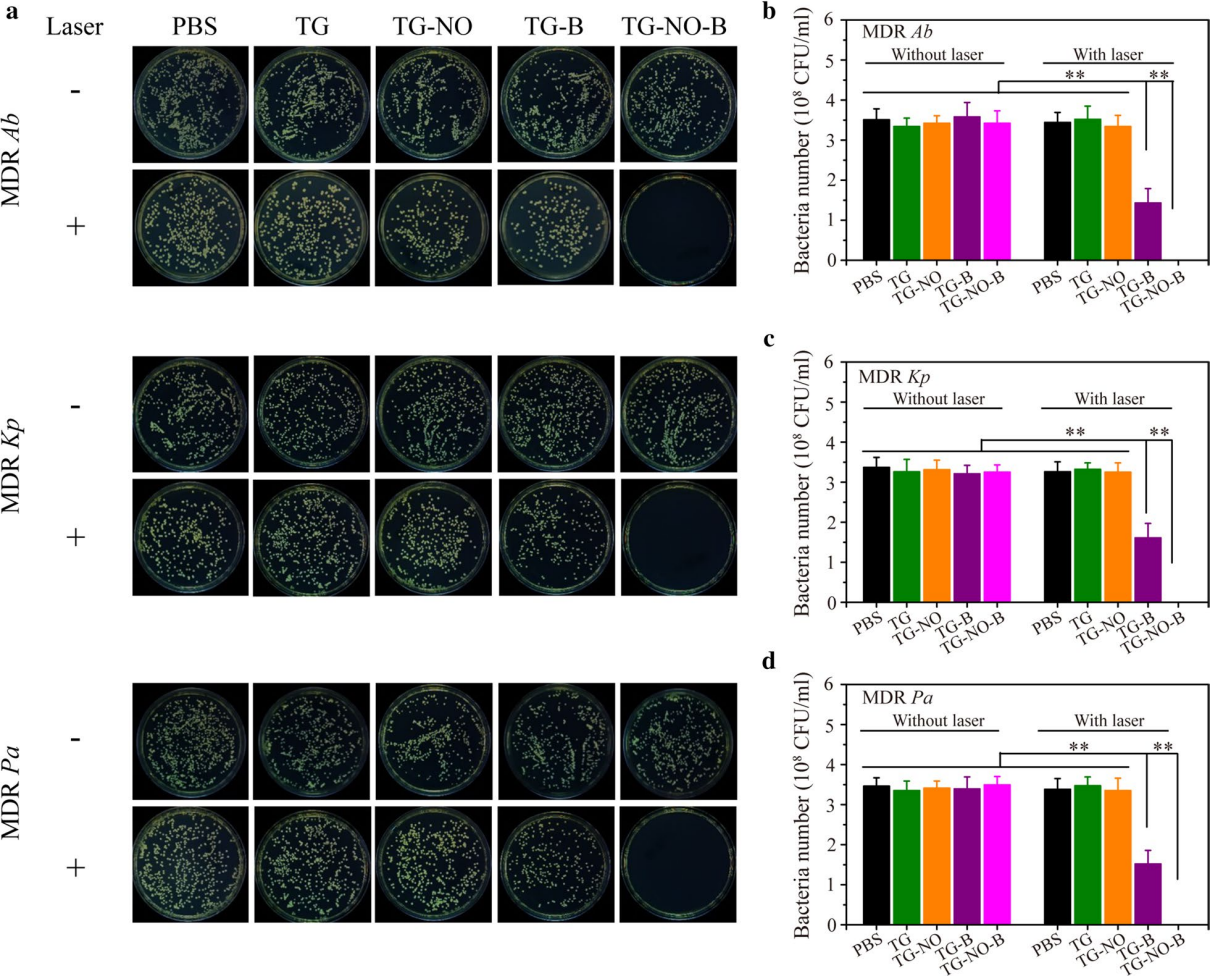
**a** Representative images of bacterial CFUs of MDR *Ab*, MDR *Kp* and MDR *Pa* biofilm exposed to PBS, TG, TG-NO, TG-B, TG-NO-B, PBS + NIR, TG +NIR, TG-NO +NIR, TG-B +NIR and TG-NO-B. Quantitative analysis of **b** MDR *Ab*, **c** MDR *Kp* and **d** MDR *Pa* biofilm receiving the treatments of PBS, TG, TG-NO, TG-B and TG-NO-B with or without NIR laser irradiation

Following demonstrating the efficacy of TG-NO-B against planktonic bacteria, the efficacy against even more refractory bacterial biofilms was further investigated by a crystal violet (CV) staining assay. The biofilm capacity (CV color intensity) of the control group (PBS) was defined as 100%. As shown in Fig. 6, upon exposure to NIR laser irradiation (0.75 W/cm^2,^ 10 min), TG-B and TG-B-NO could eliminate ~ 42% and ~ 80% of the biofilms at a concentration of 100 μg/mL, respectively. Moreover, under NIR laser irradiation, the biofilm ablation of TG-NO-B increased in a concentration-dependent manner. In contrast, without the laser irradiation, TG-NO-B exhibited no significant anti-biofilm activity, indicating the amount of NO released is too small to reach and disperse the biofilms. Furthermore, the viability of the bacteria within the biofilms was evaluated by the standard plate counting assay. As shown in Fig. 7, similar to the results of the CV staining assay, TG-NO-B with NIR laser irradiation showed a much stronger bacteria-killing activity compared to its counterparts (TG, TG-B and TG-NO) with or without the laser irradiation. In particular, under NIR laser irradiation, TG-NO-B could kill almost 100% of the bacteria within the biofilms. These results forcefully demonstrate that TG-NO-B is also a promising antibiofilm agent against MDR Gram-negative bacteria.

### In vivo biodistribution of TG-NO-B

In order to investigate the in vivo biodistribution of TG-NO-B, the TG-NO-B nanosheets were firstly labeled with the fluorescent dye CY5-PEG-SH via the covalent binding between thiol groups on CY5-PEG-SH and thiol groups on TG [38]. The successful Cy5-labeling was evidenced by the distinct absorption peak at 650 nm (Additional file 1: Fig. S9A) and fluorescent emission peak at 670 nm (Additional file 1: Fig. S9B) of the conjugated Cy5-TG-NO-B, which was consistent with the absorption and emission peak of Cy5, respectively.

**Fig. 8:**
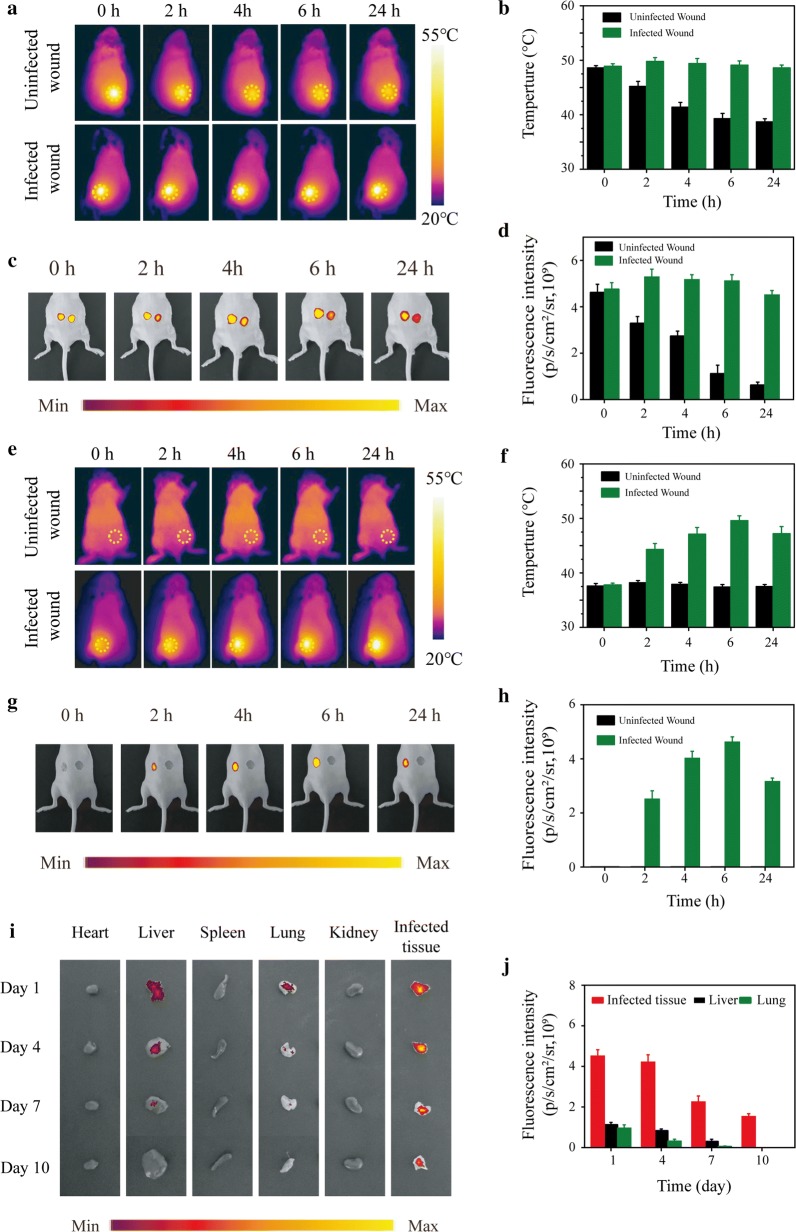
**a** Thermographic images, **b** the corresponding temperature measurements, **c** NIRF images and **d** the corresponding NIRF signal intensities of the mice with infected skin wounds after local injection of Cy5-TG-NO-B at 0, 2, 4, 6 and 24 h posttreatment. **e** thermographic images, **f** the corresponding temperature measurements, **g** NIRF images and **h** the corresponding NIRF signal intensities of the mice with infected skin wounds after intravenous injection of Cy5-TG-NO-B at 0, 2, 4, 6 and 24 h posttreatment. **i** NIRF images and the corresponding **j** NIRF signal intensities of the heart, liver, spleen, lung, kidney and the infected wound tissue extracted from the test mice intravenously injected with Cy5-TG-NO-B at the indicated time points (days 1, 4, 7, 10) postinjection

To evaluate whether the TG-NO-B could accumulate at the bacterial infection site, the in vivo NIRF and thermographic imaging were performed. As shown in Fig. 8 and Additional file 1: Fig. S10, after local or intravenous injection with Cy5-TG-NO-B, the fluorescence signal of the infected wound increased gradually with the time, peaked at 6 h post-treatment, and still remained rather high at 24 h after treatment. However, time-dependent fluorescence signal enhancement was not observed in the uninfected wound. Coinciding with the NIRF imaging results, the thermographic images demonstrated that the hyperthermia temperature in the infected wound also exhibited a time-dependent increase, while the temperature in the uninfected wound decreased continuously or did not change at all.

Furthermore, the test mice were sacrificed and the biodistribution of TG-NO-B in the major organs and infected tissues were revealed using ex vivo NIRF imaging. As shown in Fig. 8i, j, TG-NO-B maintained accumulation at the infected site for at least 10 days. Besides accumulated in the infected wound, TG-NO-B also distributed in the liver and lung, which was consistent with previous studies [51], mainly as a result of the phagocytosis of the reticuloendothelial system [52]. At 10 days post-treatment, no detected fluorescent signals were observed in the major organs, demonstrating that TG-NO-B was excreted out of the body. Moreover, as expected, similar results were found in the subcutaneous abscess (Additional file 1: Fig. S10I and J). Collectively, all the above results support that TG-NO-B can specifically target the infected site in vivo.

### In vivo antibacterial and antibiofilm activity of TG-NO-B

The in vivo antibacterial and antibiofilm effect of TG-NO-B was investigated on the murine MDR-*Pa*-infected cutaneous wound model and subcutaneous abscess model, respectively. Following the formation of infected wound or subcutaneous abscess, the test mice were locally inoculated with 100 μL of PBS, TG, TG-NO, TG-B and TG-NO-B (100 μg/mL), respectively, and the mice in the laser groups were further irradiated with an 808 nm laser (0.75 W/cm^2^, 10 min) at 6 h post-inoculation.

**Fig. 9:**
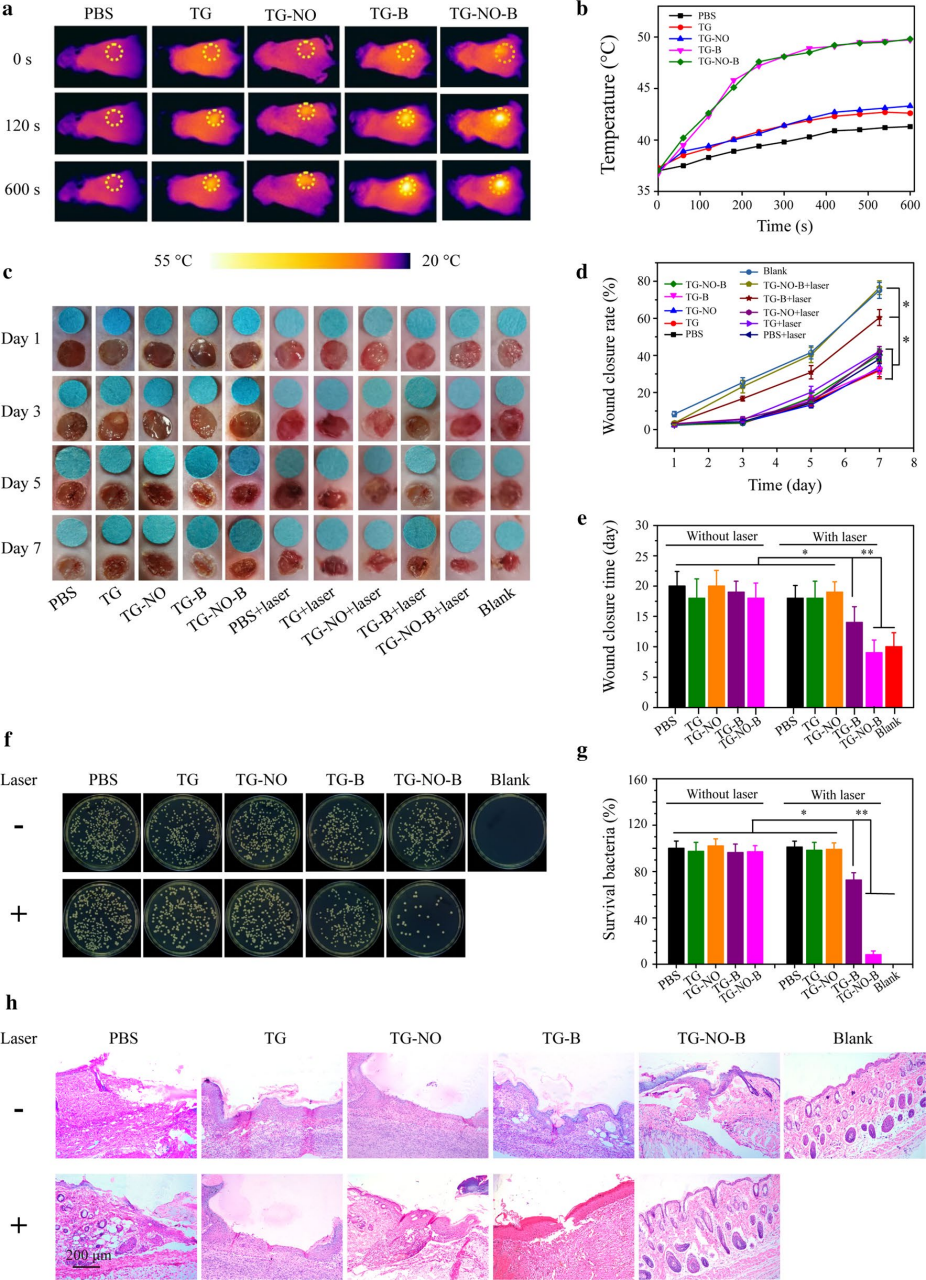
**a** Infrared thermography (0.75 W/cm^2^, 10 min) and **b** corresponding temperature curves of the mice with infected skin wounds after local injection of PBS, TG, TG-NO, TG-B and TG-NO-B at 6 h posttreatment. **c** Representative macroscopic appearances of the wounds from the PBS, TG, TG-NO, TG-B, TG-NO-B, PBS + NIR, TG + NIR, TG-NO + NIR, TG-B + NIR, TG-NO-B + NIR and blank groups. **d** The wound closure rates at different time points and **e** the wound closure times. **f** Representative images of bacterial CFUs from the wounds and **g** the corresponding bacterial viabilities. **h** Representative H&E staining images of infected skin wounds that received various treatments after 7 days

**Fig. 10:**
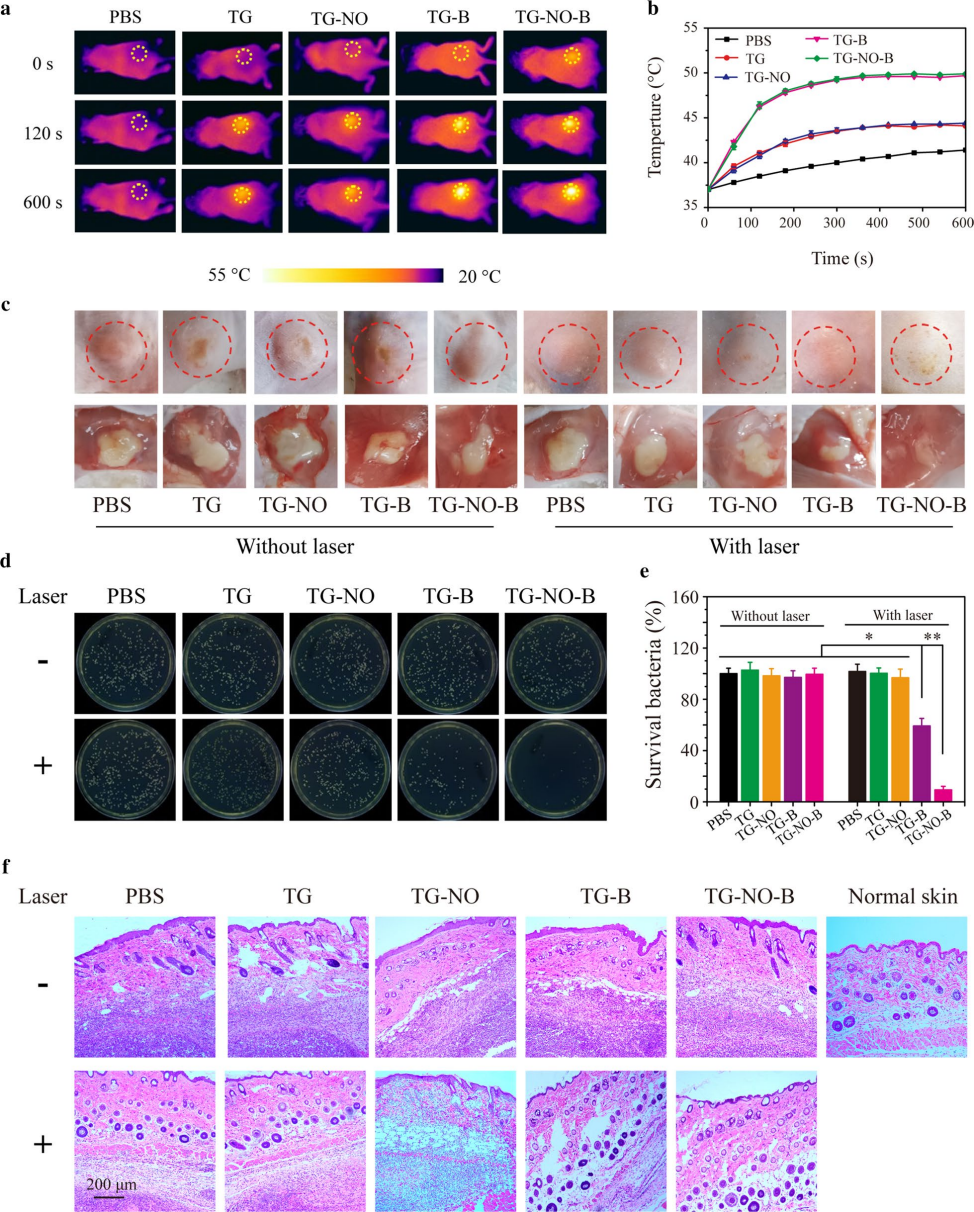
**a** Infrared thermography (0.75 W/cm^2^, 10 min) and **b** corresponding temperature curves of the mice bearing subcutaneous abscess after local injection of PBS, TG, TG-NO, TG-B and TG-NO-B at 6 h posttreatment. **c** Representative macroscopic appearances and biopsied photographs of the abscesses from the PBS, TG, TG-NO, TG-B, TG-NO-B, PBS + NIR, TG + NIR, TG-NO + NIR, TG-B + NIR and TG-NO-B + NIR groups after 12 days. **d** Representative photographs of bacterial CFUs and **e** corresponding quantitative results under various treatments. **f** Representative H&E staining images of abscesses that received various treatments

As shown in Figs. 9a and 10a, the temperature increased to ~ 49 °C and ~ 49 °C for the TG-B- and TG-NO-B-treated group, respectively; while no obvious temperature increase was observed in the PBS-treated group. Moreover, the temperature was elevated to ~ 42 °C and ~ 43 °C for the TG- and TG-NO-treated group, respectively. This result also further confirms that TG-NO-B indeed specifically target the infected site in vivo.

As shown in Fig. 9c, at 3 days post-treatment, no evident swelling and purulent exudation in the wounds were found in the TG-NO-B + NIR and blank (uninfected wounds without any treatment) groups; however, varying degrees of redness and inflammatory secretions were observed in the other groups. At 5 days post-treatment, scab formation occurred on the wound surfaces of all groups. After being treated for 7 days, obvious wound closure and newly formed epidermis were seen in the TG-NO-B + NIR and blank groups. The wound healing rate in the TG-NO-B + NIR group and the blank group was 76.5% and 75.0% (76.5% vs 75.0%, *P* = 1.00), respectively, which was much higher than that of the other groups. Moreover, the wound closure time in the TG-NO-B + NIR group and the blank group was 9 days and 10 days, respectively, which was significantly shorter than that of the other groups (Fig. 9e). In order to quantitatively analyze the in vivo antibacterial potency, at 24 h post-treatment, the infected wound tissues were collected, homogenized and spread on the agar plates. The grown bacterial colonies were photographed and counted (Fig. 9f, g). The survival rate of the control group was defined as 100%. As depicted in Fig. 9g, the bacteria survival rates in the TG, TG-NO, TG-B, TG-NO-B, PBS + NIR, TG + NIR and TG-NO + NIR groups were all similar to those in the control groups (PBS and PBS + NIR) (*P* > 0.05), while the bacteria survival rates significantly decreased to ~ 62% and ~ 7% in the TG-B + NIR group and the TG-NO-B + NIR group, respectively, indicating that TG-NO-B + NIR also showed an outstanding antibacterial effect in vivo due to synergistic chemo-PTT. Furthermore, histological analysis of the infected wounds was further conducted. As shown in Fig. 9h, perfect re-epithelialization and granulation tissue formation were clearly observed in the wounds of the TG-NO-B + NIR and blank groups, while different degrees of skin structure destruction and inflammatory infiltration existed in the other groups. All these results strongly demonstrate the synergistic effect of TG-NO-B via PTT and NO release for controlling wound infection and promote wound healing. Notably, no obvious foreign-body reaction in the healthy tissues around the wound was observed in the TG-NO-B + NIR group, suggesting no damage to ambient healthy tissues. This result also supports that TG-NO-B can specially target the infected site in vivo. Thus, the synergistic chemo-PTT of TG-NO-B is an effective and safe strategy to treat MDR Gram-negative bacteria infected wounds.

As shown in Fig. 10c, at 12 Days post-treatment, the mice in the TG-NO-B + NIR group showed no obvious abscess or inflammation on the dorsa, while different sizes of abscesses were still presented in the other groups. Moreover, the abscessed tissues were also homogenized and spread on the agar plates, followed by the bacterial colony counting. The CFU counts were standardized by the percentage of the control group (PBS). As shown in Fig. 10d, e, the treatments of TG, TG-NO, TG-B, TG-NO-B, PBS + NIR, TG + NIR and TG-NO + NIR could not eradicate the bacteria in the biofilm; whereas the bacterial survival rate in the TG-B + NIR group was reduced to ~ 59%, which revealed a moderate antibiofilm efficiency. Notably, among all the treatment groups, the TG-NO-B + NIR group showed the strongest antibiofilm activity with above 90% reduction in the bacterial viability. Furthermore, the healing of the subcutaneous abscesses with each treatment was evaluated by histological analysis. As shown in Fig. 10f, the treatment site of TG-NO-B + NIR presented a morphological feature similar to the healthy skin, and no obvious infiltration of inflammatory cells was found; whereas different degrees of subcutaneous tissue destruction and inflammatory response were still observed in the other groups. These results suggest that TG-NO-B can also serve as a superb alternative for the treatment of subcutaneous abscesses with MDR Gram-negative bacterial infections via the synergistic effect of PTT and NO release.

### In vitro and in vivo biosafety

**Fig. 11:**
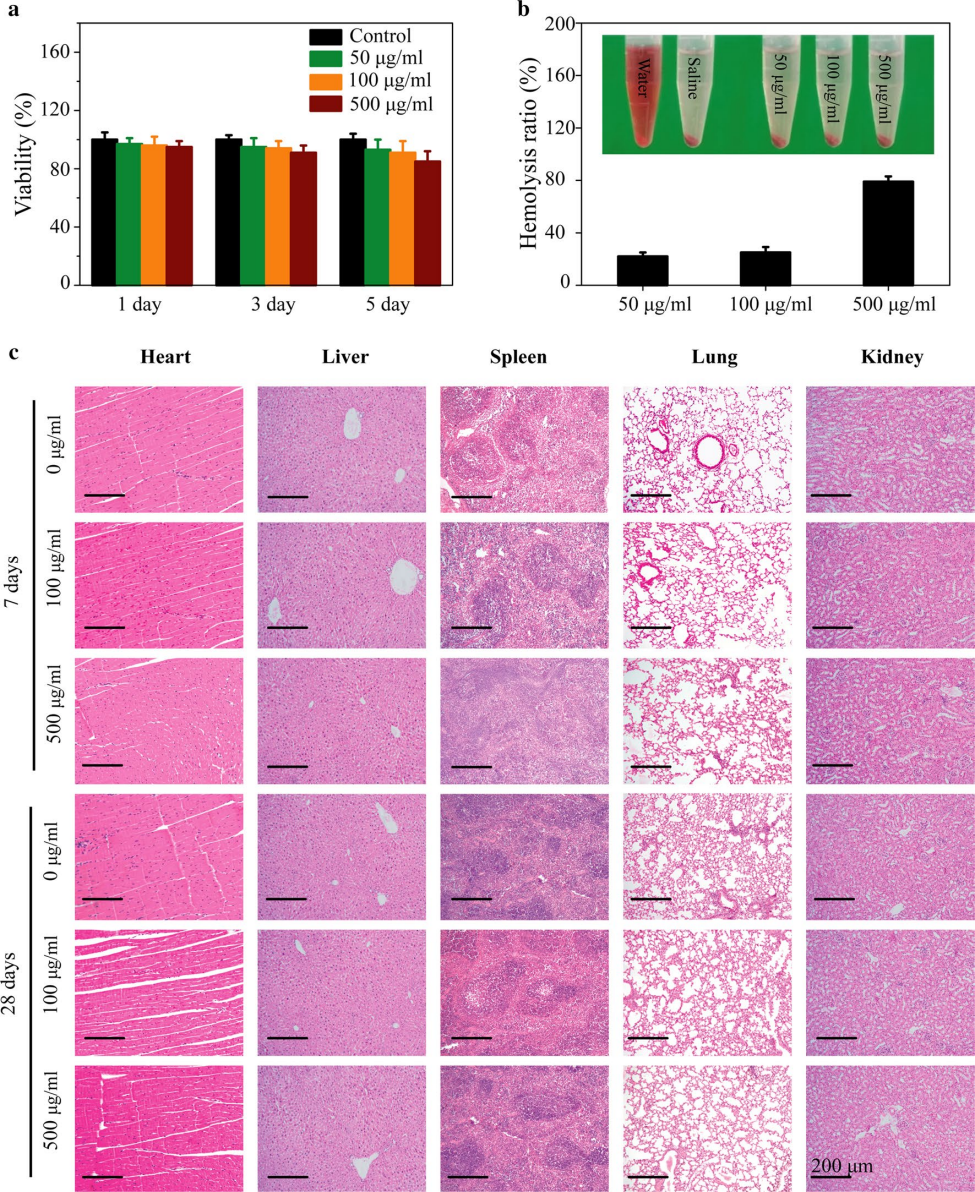
**a** 3T3 fibroblast viabilities treated with various concentrations of TG-NO-B at the indicated time points in vitro. **b** Hemocompatibilities of TG-NO-B at different concentrations. **c** H&E staining images of the major organs (heart, liver, spleen, lung, and kidney) from the mice 7 days and 28 days after intravenous injection of PBS or TG-NO-B at doses of 100 and 500 μg/mL

Considering the nanotoxicity towards skins and the possibility of practical application [53], it is really essential to investigate the biocompatibility of TG-NO-B. Skin cells (e.g. 3T3 fibroblasts) and blood cells (e.g. red blood cells), are most commonly used as model cells for the cytotoxicity/hemocompatibility evaluation of nanomaterials [54]. First, to assess the in vitro cytotoxicity of TG-NO-B, its effect on the viability of 3T3 fibroblasts was detected using the CCK-8 assay. As shown in Fig. 11a, after co-incubation with TG-NO-B (0–500 μg/mL) for 5 days, TG-NO-B had negligible influence on the viabilities of the cells. Moreover, the survival rate of the cells was still over 88.6% even at the high concentration of 500 μg/mL and 5 days of incubation. Next, the hemocompatibility of TG-NO-B was evaluated using the hemolysis test. After incubation with TG-NO-B (0–500 μg/mL) for 3 h, no obvious hemolysis of human erythrocytes was observed, while the deionized water-treated group as the positive control exhibited sever hemolysis (Fig. 11b). Moreover, the hemolysis ratio was less than 1% even at a high concentration of 500/μg/mL (Fig. 11b).

Furthermore, the in vivo potential biosafety of TG-NO-B was also assessed. As shown in Fig. 11c, no significant pathological abnormalities of the major organs (heart, liver, spleen, lung and kidney) were observed at 7 and 28 days after intravenous injection of TG-NO-B (100 μg/mL and 500 μg/mL), which suggested that TG-NO-B exerted negligible influence on the test mice in the in vivo experiments. Moreover, 28 days post-treatment, the routine blood test (white blood cells, red blood cells, hemoglobin and blood platelets), liver function markers (ALT, AST), kidney function markers (BUN, Cr) and cardiac enzymes (CK, CK-MB) were also examined and found to be all within the normal reference ranges (Additional file 1: Fig. S11), indicating that TG-NO-B had no obvious damages to the hematological system and hepatic, renal and cardiac functions. Taken together, these results reveal that TG-NO-B exhibits excellent biocompatibility both in vitro and in vivo and can be utilized as a promising candidate for biomedical applications.

In the past few years, increasing evidences have demonstrated that graphene and its derivatives can exhibit toxic effects at the molecular, cellular, organ or animal levels. Furthermore, the toxicity of graphene and its derivatives depends on a lot of factors, e.g. dose, exposure duration, exposure route, size, shape, number of layers, charge, and surface properties, etc. [55–57]. For example, Feng et al. [58] revealed that the viability of PC12 cells was decreased in a dose- and time-dependent manner following graphene oxide (GO) exposure. Additionally, GO triggered an increased autophagic response and the impairment of autophagic flux via disrupting lysosome degradation capability. Importantly, the elevated level of p62 protein was functionally involved in cell apoptosis via the caspase-9 dependent pathway. Chatterjee et al. [59] reported that graphene nanoplatelets [GNPs—pristine, carboxylate (COOH) and amide (NH_2_)] exhibited higher toxicity than GOs [single layer (SLGO) and few layers (FLGO)] in Beas2B cells, and among the GNPs, the order of toxicity was pristine > NH_2_ > COOH, which was maintained in the reproductive toxicity of *C. elegans.* However, GOs were found to be more toxic in the worms than GNPs. Moreover, SLGO exhibited profoundly greater dose dependency than FLGO in both systems. Jia et al. [60] found that the small-sized materials at the lower concentrations showed more toxic potential to reduce cell viability and increase DNA damage in HEK 293T cells, compared to the medium and large sizes of graphene (G) and GO. Moreover, GO elevated DNA damage in cells and also disturbed the expressions of related genes in cells and zebrafish, while G showed more obvious effects on cell viability. Nevertheless, the toxicity of graphene and its derivatives can be greatly mitigated via surface functionalization with some macromolecules, such as polyethylene glycol (PEG), chitosan or serum proteins [9, 61, 62]. Here, TG-NO-B showed excellent in vitro and in vivo biocompatibility, most probably due to the boronic acid surface functionality [35, 63], the improved selectivity towards bacterial cells over mammalian cells and the relatively rapid excretion.

To date, some researches have been conducted on PTT/PDT/NO-based antibacterial platforms. Compared to the earlier studies, our present work has much more advantages. First, unlike spherical/rod-like or square-shaped NO-releasing nanoparticles [1, 22–24], TG-NO-B nanosheets may ensure more effective contact with bacterial cells or biofilms due to its special 2D-strcurute and large surface area [19]. Second, compared with surface charge switchable nanoassemblies [19, 22, 25, 26], TG-NO-B may exhibit a more specific and strong binding affinity to Gram-negative bacteria and their biofilms via the covalent coupling. Both of these superiorities may largely enhance the antibacterial efficiency and minimize the undesirable damages to ambient healthy tissues. Third, different from previously reported boronic acid functionalized nanomaterials [4, 21], the bacteria/biofilm-targeting and antibacterial capacities of TG-NO-B are fully confirmed both in vitro and in vivo. Fourth, in contrast with single PTT platforms [19, 27], with one-step operation, single NIR light can trigger hyperthermia and controllable NO release of TG-NO-B simultaneously, which can synergistically disrupt bacterial cell membrane, further cause leakage and damage of intracellular components, and finally induce bacteria death. Furthermore, the bacterial cell membrane destruction can improve the permeability and sensitivity to heat, decrease the photothermal temperature and avoid damages caused by high temperature.

## Conclusions

To sum up, we have successfully developed a multifunctional platform (TG-NO-B) for simultaneous PTT and NO release triggered by a single NIR light source. The as-designed antibacterial depot could specially target and accumulate the sites of Gram-negative bacteria-associated infections via the covalent coupling effect, and showed an outstanding chemo (NO)-photothermal (TG) synergistic therapeutic effect on the infected wound and subcutaneous abscess, resulting in the effective elimination of bacteria and biofilms, enhancement of wound healing, and mitigation of damages to the ambient healthy tissues. Moreover, TG-NO-B exhibited excellent biocompatibility both in vitro and in vivo. Given all these apparent advantages, TG-NO-B fabricated in the present study can be considered as a promising alternative for treating infections caused by MDR Gram-negative bacteria and their biofilms.

## Supplementary information

**Additional file 1.** Additional figures.

## Data Availability

All data generated or analyzed during this study are included in the article and additional file.
