# Correction to: A multifunctional platform with single-NIR-laser-triggered photothermal and NO release for synergistic therapy against multidrug-resistant Gram-negative bacteria and their biofilms

**DOI:** 10.1186/s12951-020-00689-0

**Published:** 2020-09-25

**Authors:** Baohua Zhao, He Wang, Wenjing Dong, Shaowen Cheng, Haisheng Li, Jianglin Tan, Junyi Zhou, Weifeng He, Lanlan Li, Jianxiang Zhang, Gaoxing Luo, Wei Qian

**Affiliations:** 1 Institute of Burn Research, State Key Laboratory of Trauma, Burn and Combined Injury, Key Laboratory of Disease Proteomics of Chongqing, Southwest Hospital, Third Military Medical University (Army Medical University), Chongqing, 400038 China; 2 Department of Trauma Centre, The First Affiliated Hospital, Hainan Medical University, Haikou, 570102 Hainan China; 3 Department of Pharmaceutics, College of Pharmacy, Third Military Medical University (Army Medical University), Chongqing, 400038 China

## Correction to: J Nanobiotechnol (2020) 18:59 10.1186/s12951-020-00614-5

Following publication of the original article [1], the authors were sorry to find that some photos of the agar plates were inadvertently duplicated in Fig. 7a, due to our carelessness during organizing of the large number of related images. After thorough inspection on our original data and reanalyzing the whole set of digital photos of agar plates for Fig. 7a, we confirmed that the image errors did not influence the quantitative results for the bacterial number counting, shown in Fig. 7b–d. The authors also regret that the images in Fig. 9h [TG-NO-B Laser (+)], Fig. 10f [PBS Laser (−), TG-NO Laser (−), TG-B Laser (−)] and Fig. 11c (Liver, 28 days, 0 mg/ml; Spleen, 28 days, 0 mg/ml) were mistakenly used due to the authors’ carelessness. These corrections have not changed the description, interpretation, or the original conclusions of the manuscript. The revised Figs. 7a, 9h, 10f and 11c are published in this correction.

**Fig. 7:**
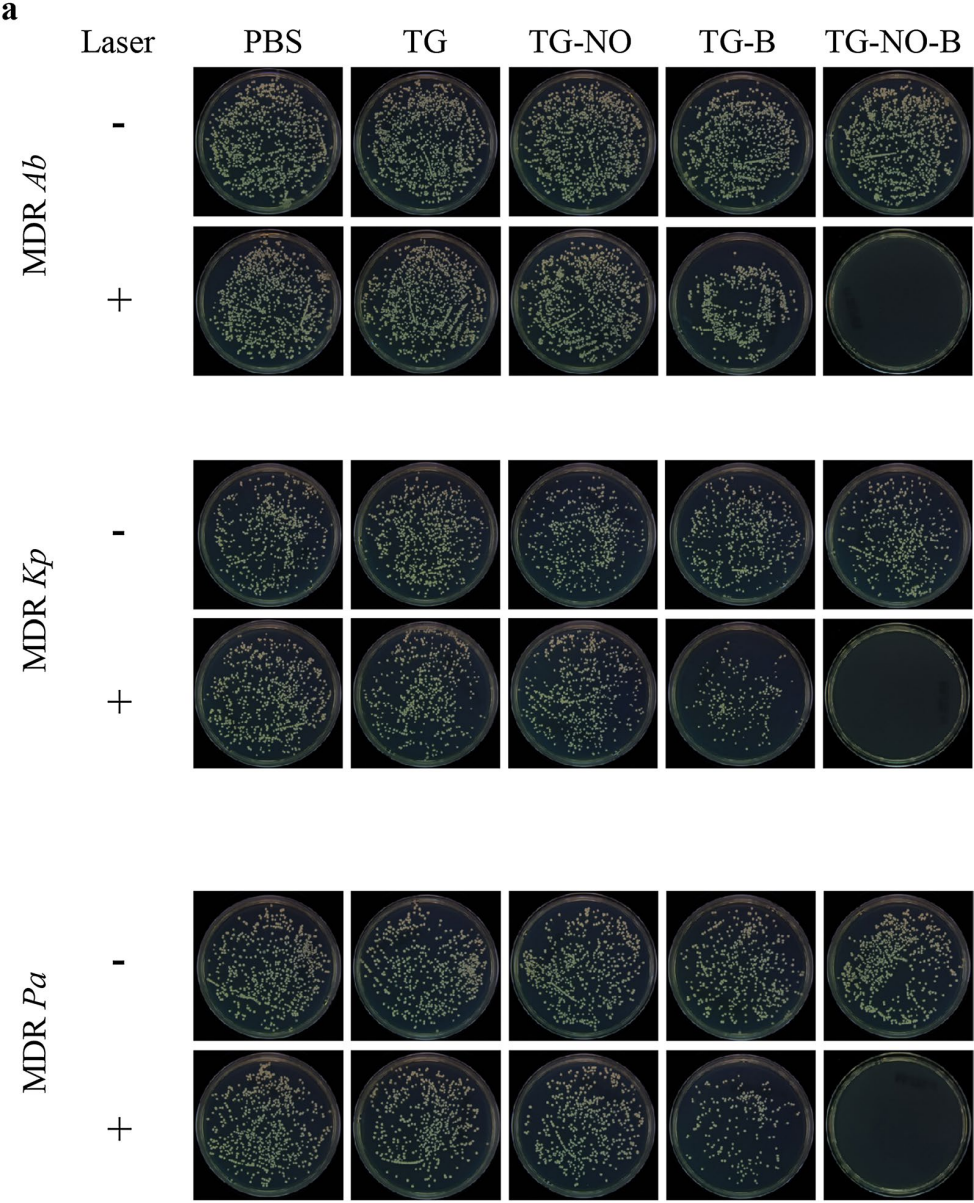
**a** Representative images of bacterial CFUs of MDR Ab, MDR Kp and MDR Pa biofilm exposed to PBS, TG, TG-NO, TG-B, TG-NO-B, PBS + NIR, TG + NIR, TG-NO + NIR, TG-B + NIR and TG-NO-B

**Fig. 9:**
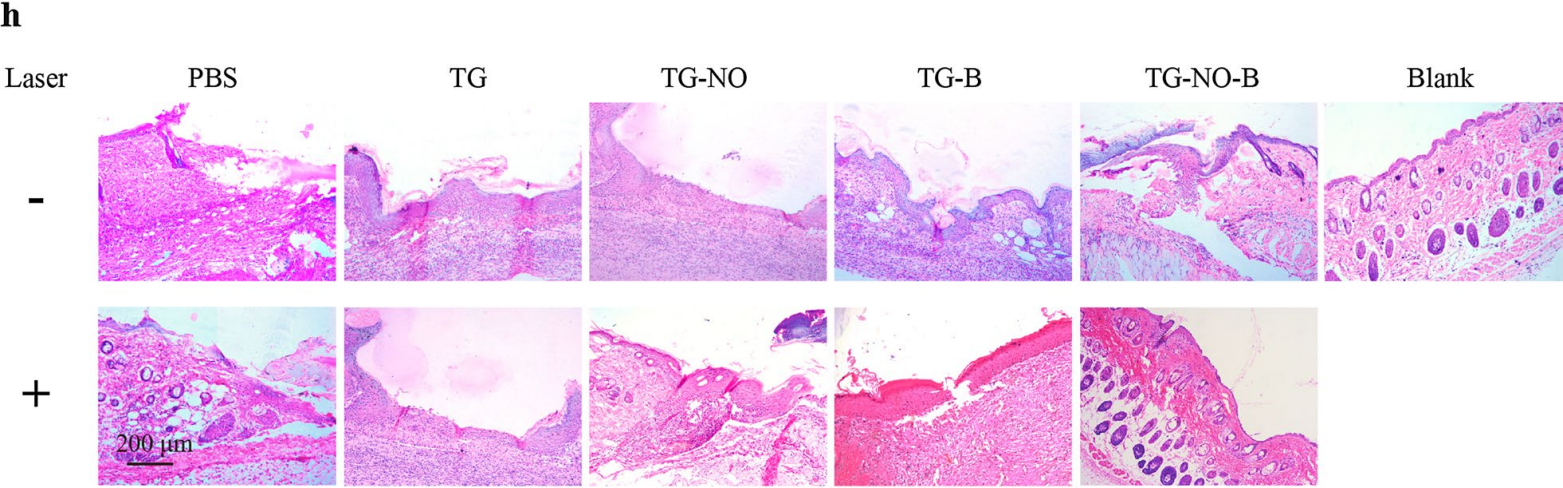
**h** Representative H&E staining images of infected skin wounds that received various treatments after 7 days

**Fig. 10:**
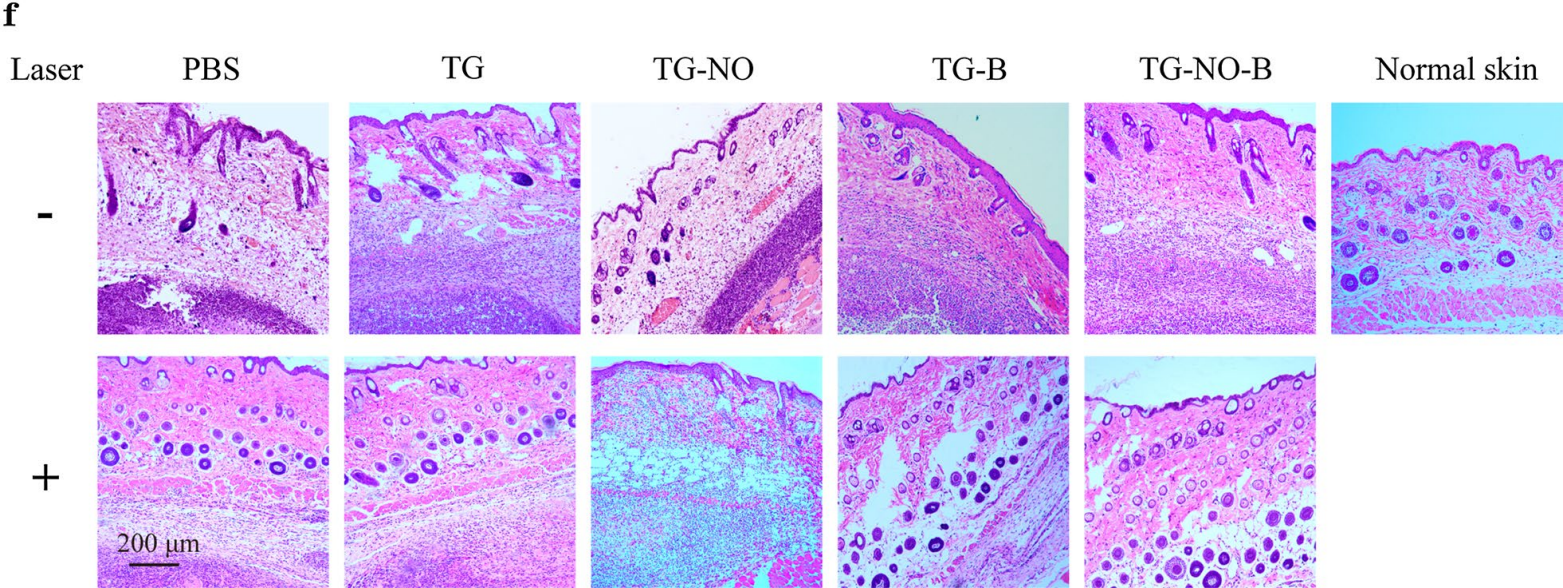
**f** Representative H&E staining images of abscesses that received various treatments

**Fig. 11:**
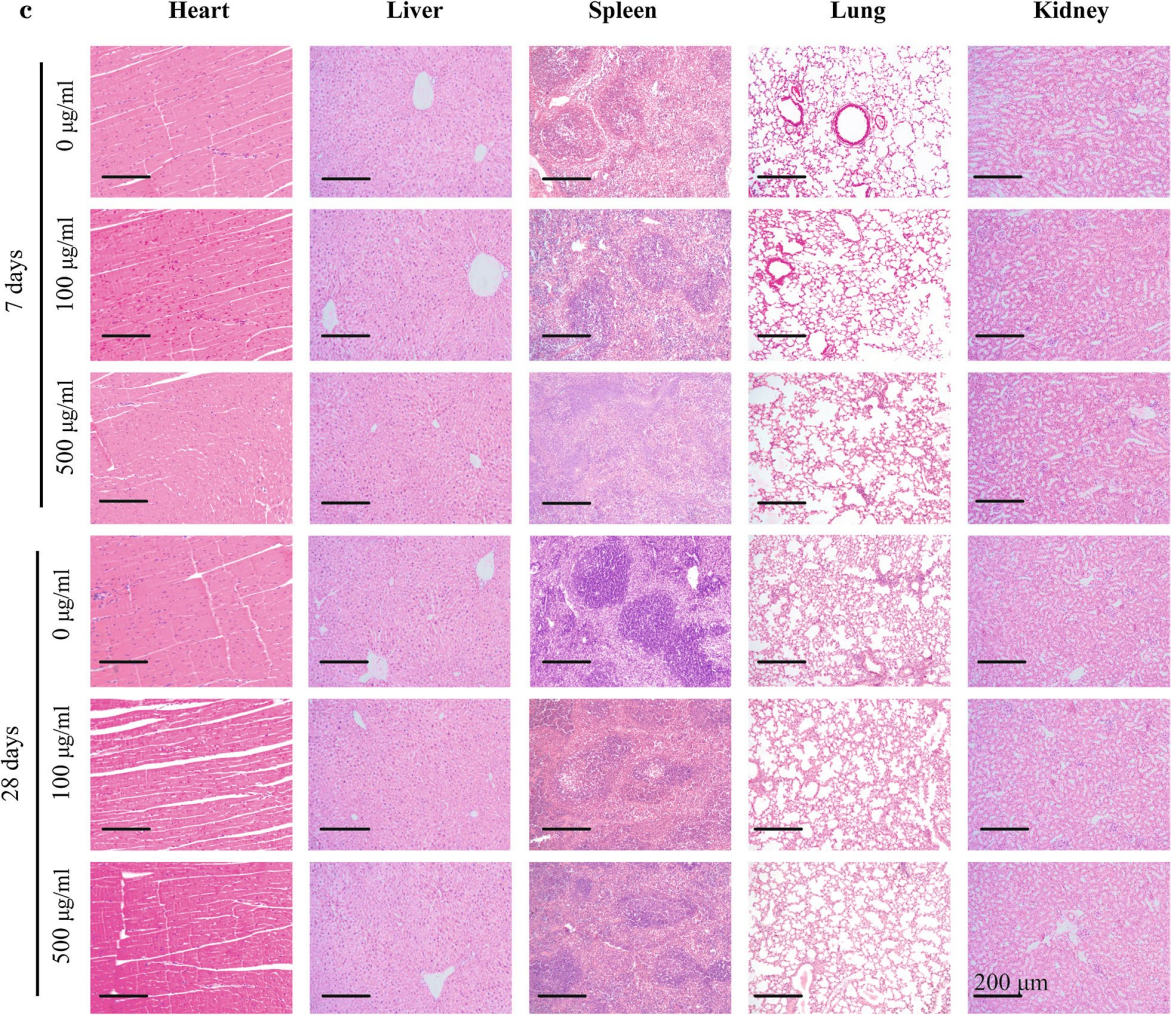
**c** H&E staining images of the major organs (heart, liver, spleen, lung, and kidney) from the mice 7 days and 28 days after intravenous injection of PBS or TG-NO-B at doses of 100 and 500 μg/mL

The authors apologize for not noticing these errors prior to publication, and for any inconvenience caused.
