# Supplementary material for: A multifunctional platform with single-NIR-laser-triggered photothermal and NO release for synergistic therapy against multidrug-resistant Gram-negative bacteria and their biofilms
Source: J Nanobiotechnology. 2020 Apr 15;18:59. doi: 10.1186/s12951-020-00614-5 (PMC7158002; doi:10.1186/s12951-020-00614-5)
Supplement: Supplementary file 1 — Additional file 1. Additional figures. [file 12951_2020_614_MOESM1_ESM.docx]

**Additional file**

**A multifunctional platform with single-NIR-laser-triggered photothermal and NO release for synergistic therapy against multidrug-resistant Gram-negative bacteria and their biofilms**

Baohua Zhao^a†^, He Wang^a†^, Wenjing Dong^a†^, Shaowen Cheng^b^, Haisheng Li^a^, Jianglin Tan^a^, Junyi Zhou^a^, Weifeng He^a^, Lanlan Li^c^, Jianxiang Zhang^c^, Gaoxing Luo^a*^, Wei Qian^a*^

a Institute of Burn Research, State Key Laboratory of Trauma, Burn and Combined

Injury, Key Laboratory of Disease Proteomics of Chongqing, Southwest Hospital,

Third Military Medical University (Army Medical University), Chongqing 400038,

China

b Department of Trauma Centre, The First Affiliated Hospital, Hainan Medical University, Haikou 570102, Hainan Province, China

c Department of Pharmaceutics, College of Pharmacy, Third Military Medical University (Army Medical University), Chongqing 400038, China

† These authors contributed equally to this work.

*Corresponding authors. Tel./fax: +86-23-68752688. E-mail address: [logxw@hotmail.com](mailto:logxw@hotmail.com); weiqian87@126.com.

**
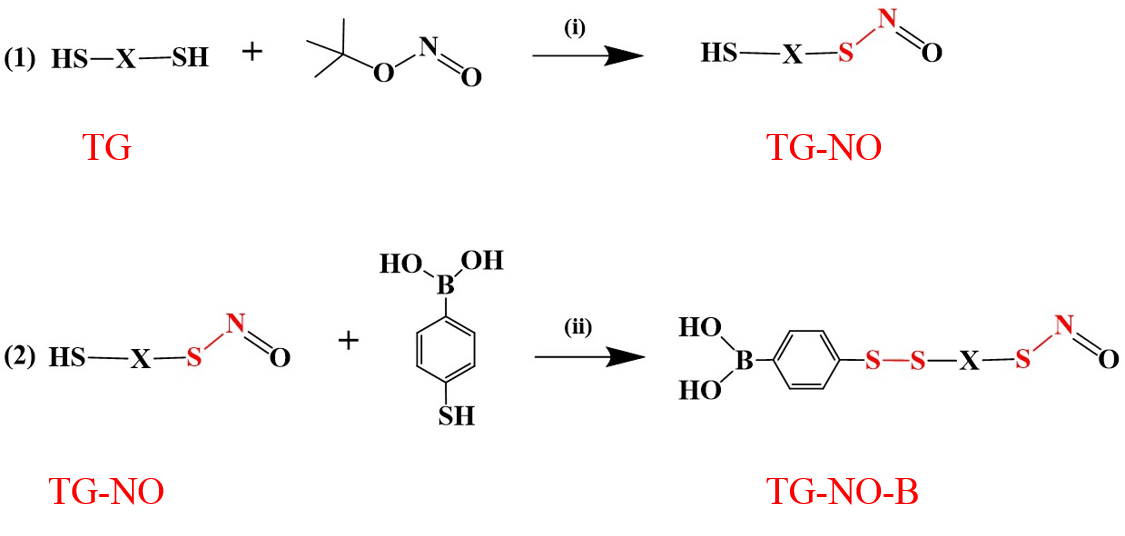
**

**Fig. S1** Synthetic route of thiolated graphene based nitric oxide nanogenerators (TG-NO) and boronic acid functionalized thiolated graphene based NO nanogenerators (TG-NO-B).


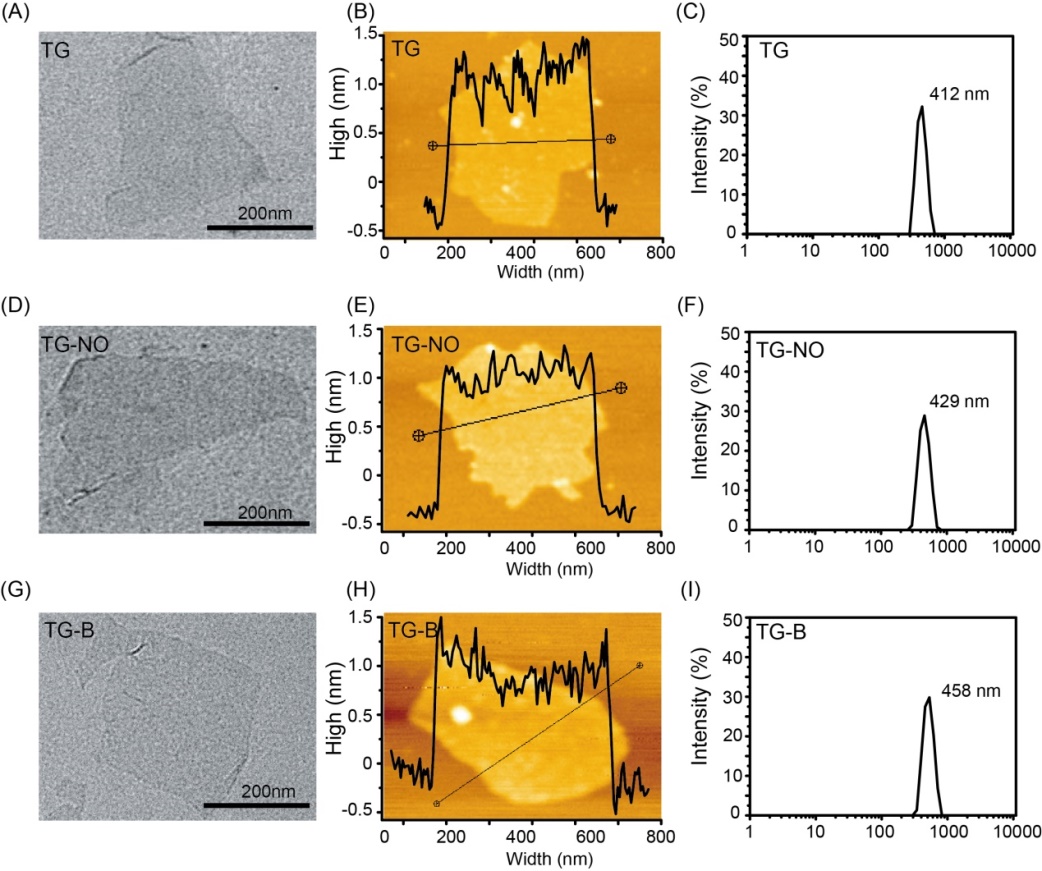


**Fig. S2** TEM images of (A) TG, (D) TG-NO and (G) TG-NO-B. AFM analysis of (B) TG, (E) TG-NO and (H) TG-NO-B. Hydrodynamic diameters (C) TG, (F) TG-NO and (I) TG-NO-B in PBS buffer measured by DLS.


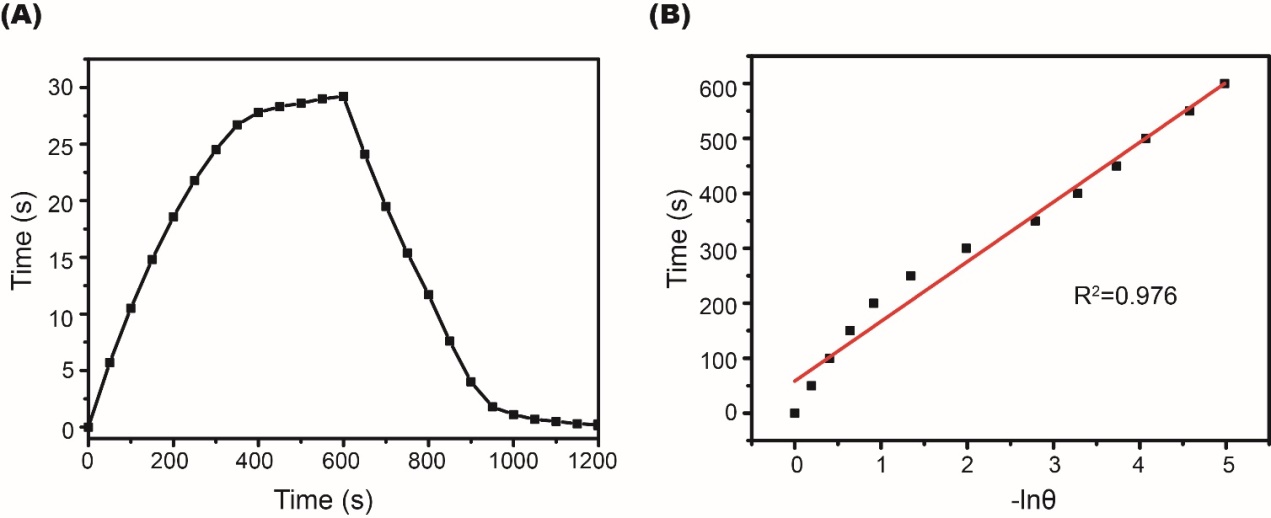


**Fig. S3** (A) Photothermal effect of the irradiation of the aqueous dispersion of TG-NO-B with the NIR laser (808 nm, 0.75 W/cm^2^), in which the irradiation lasted for 10min, and then the laser was turned off. (B) Time constant for heat transfer from the system is determined by applying the linear time data from the cooling period (after 600 s) versus negative natural logarithm of driving force temperature.


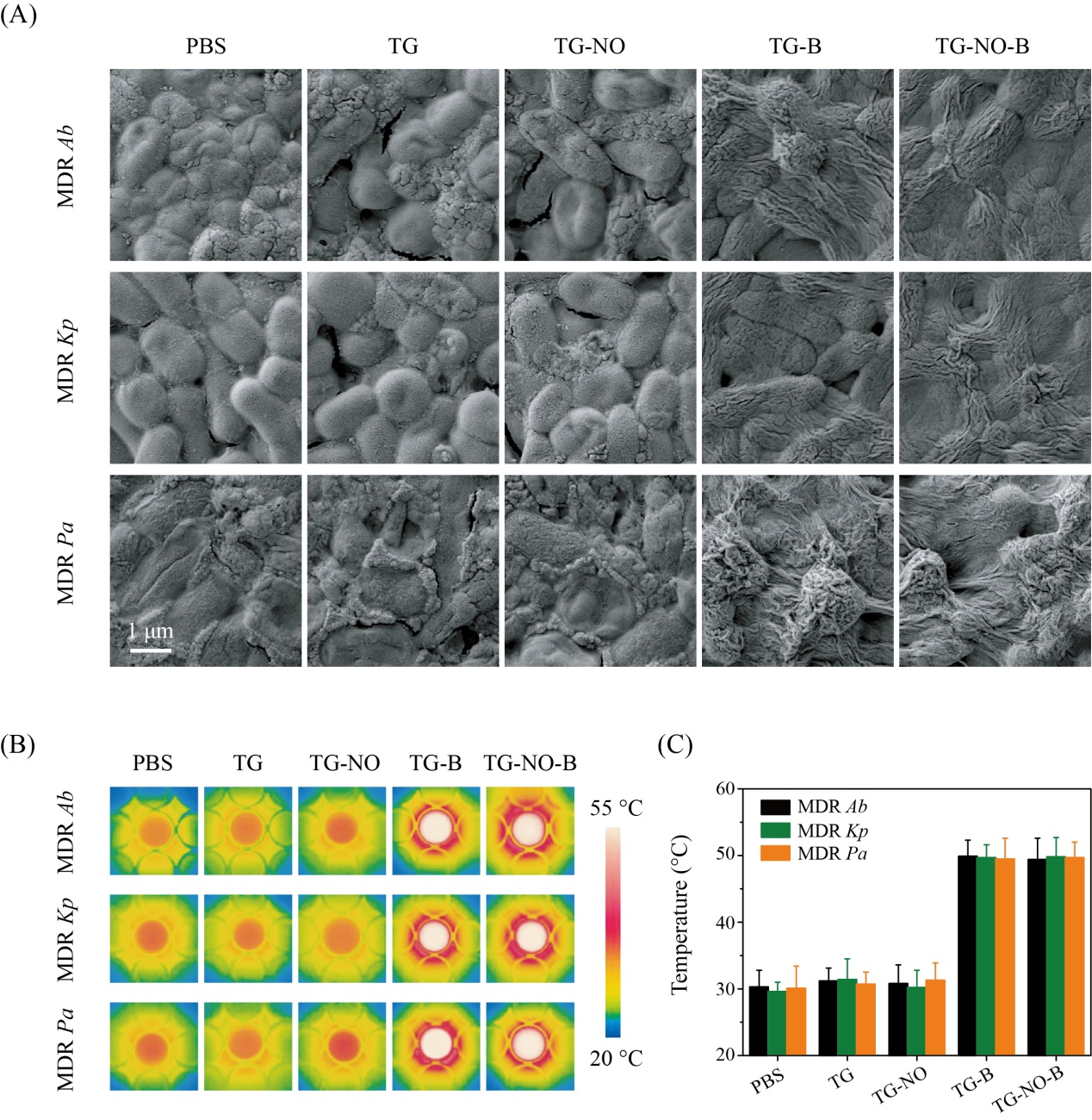


**Fig. S4** (A) Representative SEM images of MDR *Ab*, MDR *Kp* and MDR *Pa* biofilms after incubation with PBS, TG, TG-NO, TG-B and TG-NO-B. (B) Temperature measurements and (C) the corresponding thermographic images of MDR *Ab*, MDR *Kp* and MDR *Pa* biofilms after incubation with PBS, TG, TG-NO, TG-B and TG-NO-B under NIR irradiation (808nm, 0.75 W/cm^2^) for 10 min.


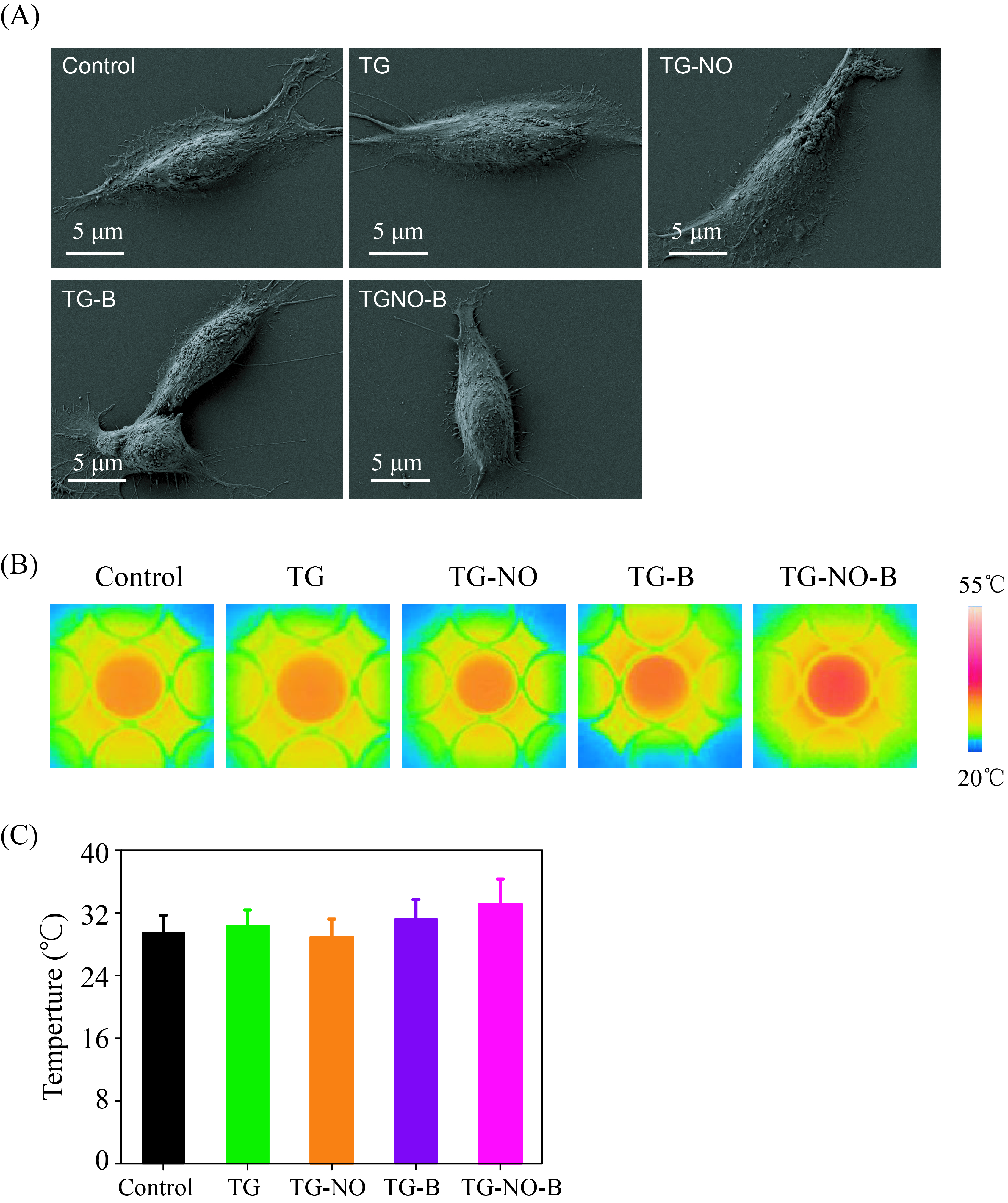


**Fig. S5** (A) Representative SEM images, (B) thermographic images and (C) temperature measurements of 3T3 fibroblasts after incubation with PBS, TG, TG-NO, TG-B and TG-NO-B under NIR irradiation (10 min, 0.75 W/cm^2^).


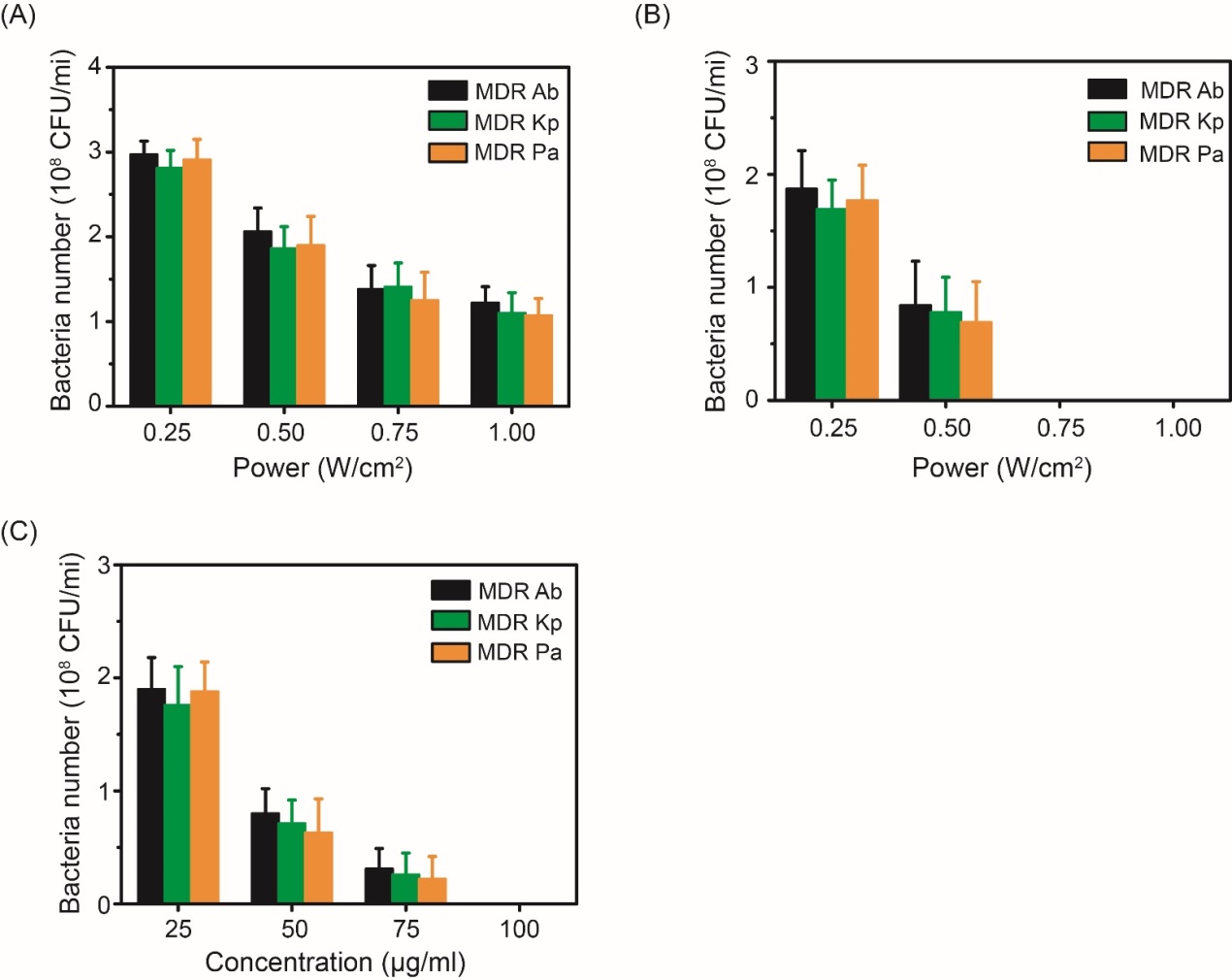


**Fig. S6** (A) Bacteria number of MDR *Ab*, MDR *Kp* and MDR *Pa* treated with TG-B (100 μg/mL) under NIR irradiation of different power densities. (B) Bacteria number of MDR *Ab*, MDR *Kp* and MDR *Pa* treated with TG-NO-B (100 μg/mL) under NIR irradiation of different power densities. (C) Bacteria number of MDR *Ab*, MDR *Kp* and MDR *Pa* treated with TG-NO-B of different concentrations under NIR irradiation (10 min, 0.75 W/cm^2^).


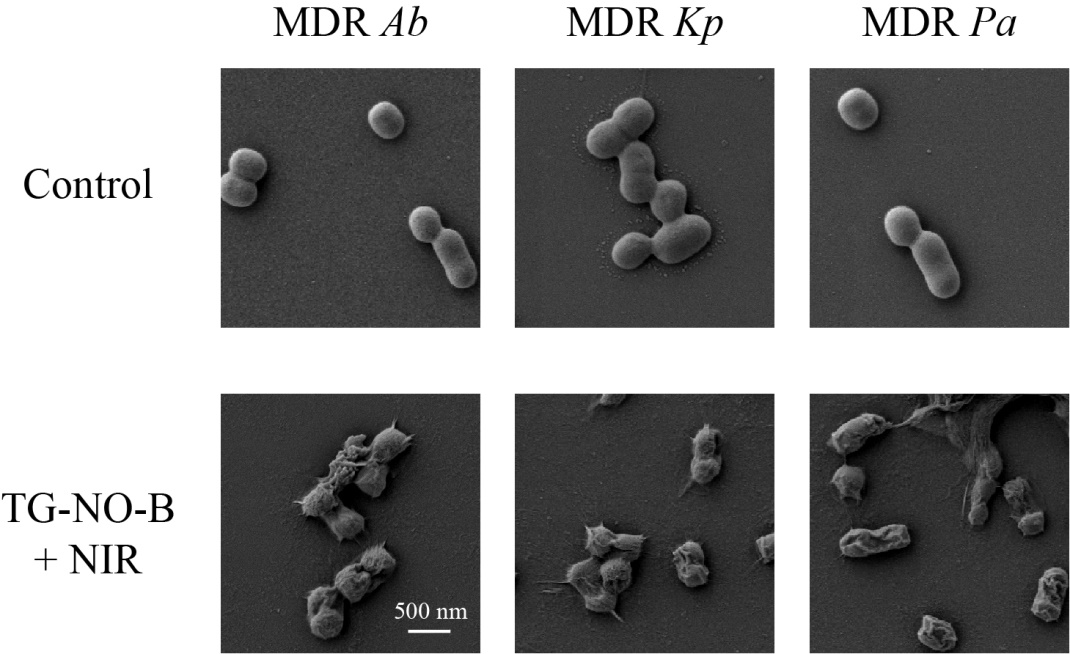


**Fig. S7** SEM images of bacteria treated with TG-NO-B under laser irradiation.


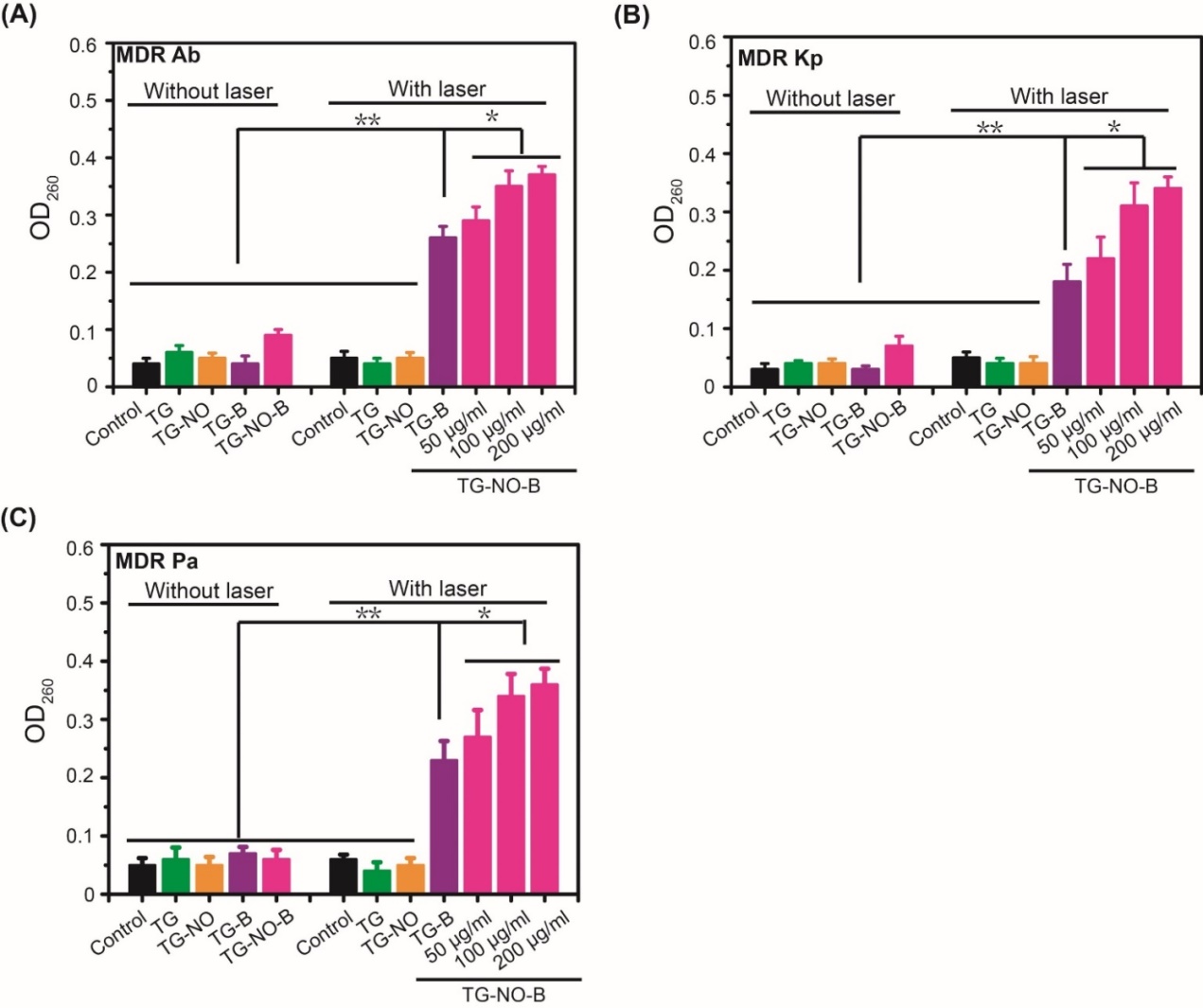


**Fig. S8** The absorbances of effluxes of bacterial cytoplasmic contents (DNA and RNA) at 260 nm after various treatments.


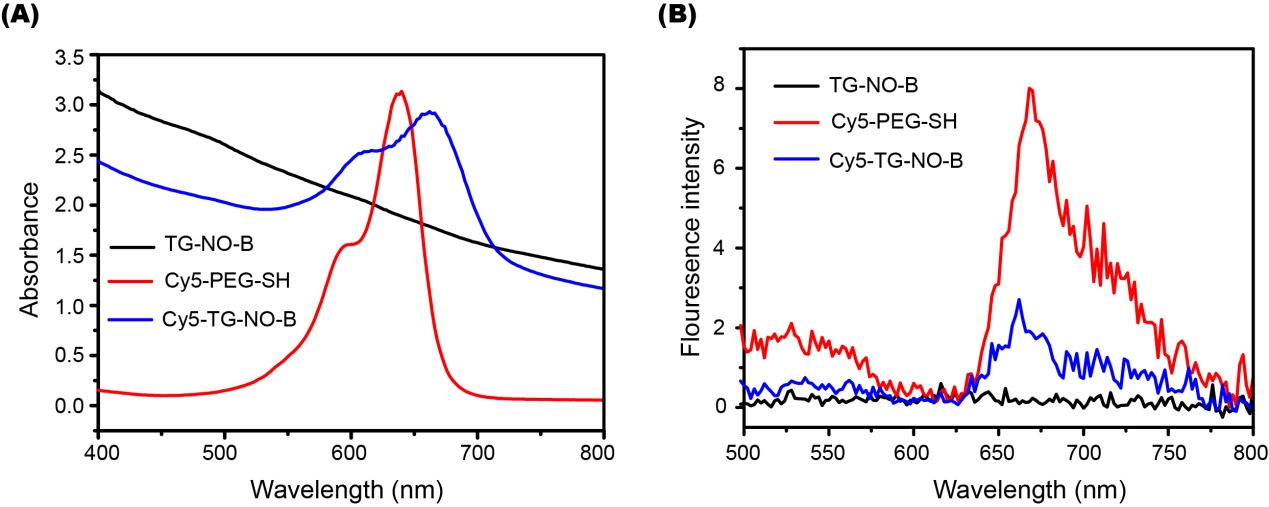


**Fig. S9** (A) UV-vis-NIR and (B) fluorescence spectra of TG-NO-B, Cy5-PEG-SH and Cy5-TG-NO-B, respectively.


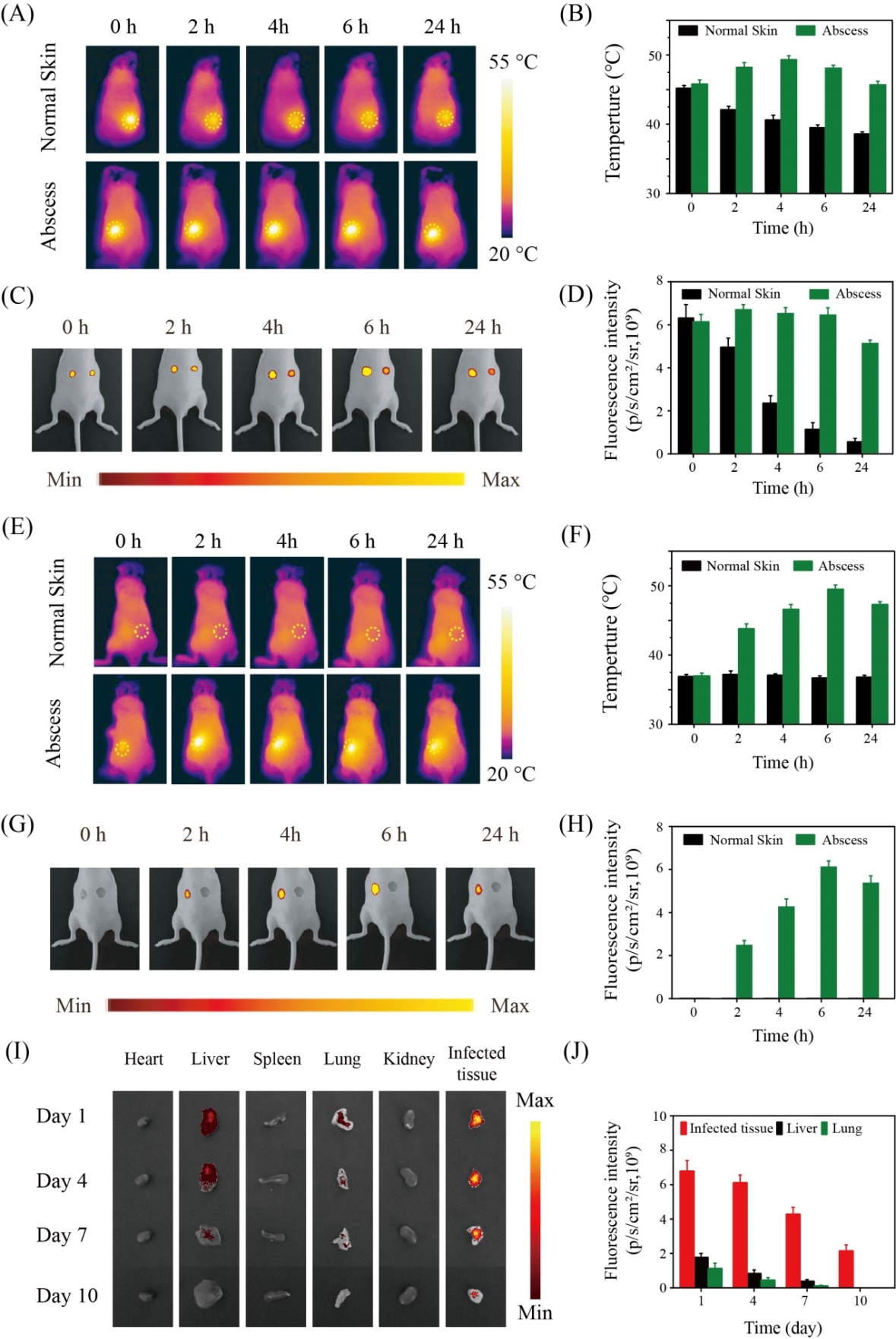


**Fig. S10** (A) thermographic images, (B) the corresponding temperature measurements, (C) NIRF images and (D) the corresponding NIRF signal intensities of the mice bearing subcutaneous abscess after local injection of Cy5-TG-NO-B at 0, 2, 4, 6 and 24 h posttreatment. (E) thermographic images, (F) the corresponding temperature measurements, (G) NIRF images and (H) the corresponding NIRF signal intensities of the mice bearing subcutaneous abscess after intravenous injection of Cy5-TG-NO-B at 0, 2, 4, 6 and 24 h posttreatment. (I) NIRF images and the corresponding (J) NIRF signal intensities of the heart, liver, spleen, lung, kidney and the abscess tissue extracted from the test mice intravenously injected with Cy5-TG-NO-B at the indicated time points (days 1, 4, 7, 10) postinjection.


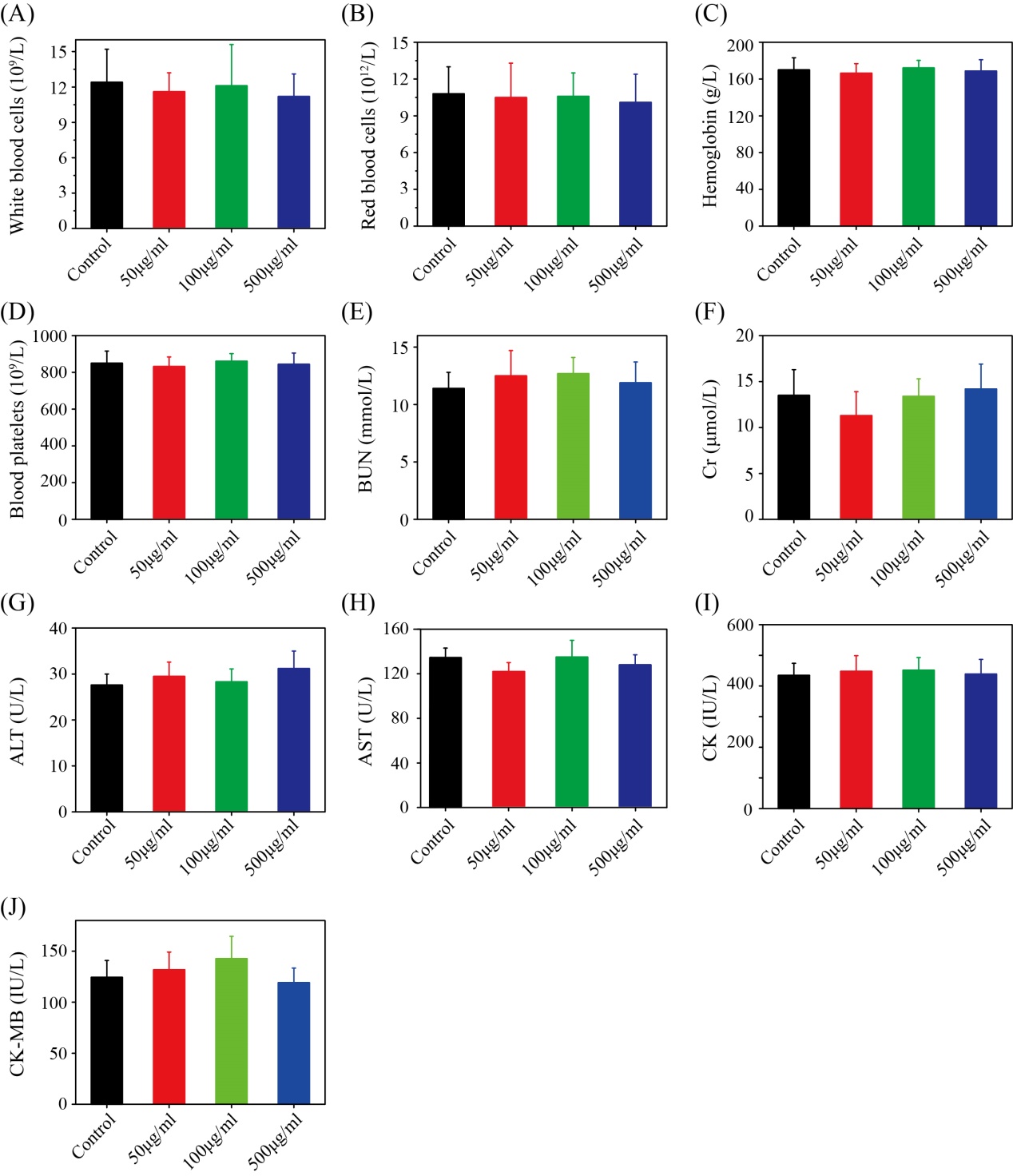


**Fig. S11** Hematology and blood biochemistry analysis of mice sacrificed at 28 days after intravenous injection of PBS or TG-NO-B at doses of 50, 100 and 500μg/mL.
